# Implementing a Doping Approach for Poly(methyl methacrylate) Recycling in a Circular Economy

**DOI:** 10.1021/jacs.3c13223

**Published:** 2024-02-21

**Authors:** Mason T. Chin, Tiangang Yang, Kevin P. Quirion, Christina Lian, Peng Liu, Jie He, Tianning Diao

**Affiliations:** † Department of Chemistry, New York University, 100 Washington Square East, New York, New York 10003, United States; ‡ Department of Chemistry, University of Connecticut, Storrs, Connecticut 06269, United States; § Department of Chemistry, University of Pittsburgh, Pittsburgh, Pennsylvania 15260, United States

## Abstract

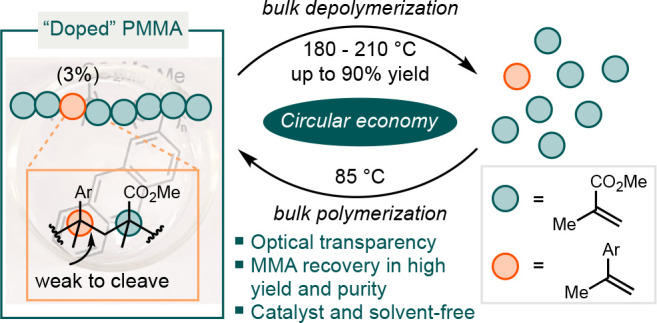

To mitigate pollution by plastic waste, it is paramount to develop polymers with efficient recyclability while retaining desirable physical properties. A recyclable poly(methyl methacrylate) (PMMA) is synthesized by incorporating a minimal amount of an α-methylstyrene (AMS) analogue into the polymer structure. This P(MMA-*co*-AMS) copolymer preserves the essential mechanical strength and optical clarity of PMMA, vital for its wide-ranging applications in various commercial and high-tech industries. Doping with AMS significantly enhances the thermal, catalyst-free depolymerization efficiency of PMMA, facilitating the recovery of methyl methacrylate (MMA) with high yield and purity at temperatures ranging from 150 to 210 °C, nearly 250 K lower than current industrial standards. Furthermore, the low recovery temperature permits the isolation of pure MMA from a mixture of assorted common plastics.

Poly(methyl methacrylate) (PMMA), commonly known as Plexiglas, is a synthetic material widely used in construction, automobiles, and electronics.^1^ PMMA, known for its transparency, serves as a cost-effective, lightweight, and shatterproof alternative to glass. The demand for PMMA experienced a recent surge during the COVID era due to its application in transparent protective barriers in public spaces.^2^ Currently, nearly 90% of PMMA ends up in landfills—a trend expected to rise as the demand for plastics continues to grow.^3,4^ While mechanical recycling offers a straightforward method for managing PMMA waste, it necessitates rigorous sorting and often results in diminished quality after multiple recycling processes.^5^

The chemical recycling of PMMA back to its monomer, methyl methacrylate (MMA), presents a sustainable solution toward a circular economy (Scheme 1A).^6−8^ PMMA is conventionally produced from MMA via radical polymerization at temperatures ranging from 50 to 90 °C.^9^ This reversible process can be tuned to favor depolymerization at elevated temperatures, which is proposed to be thermally initiated through a series of chain-scission events.^10−14^ Common pyrolysis conditions typically require temperatures above 400 °C to achieve full recovery of MMA from PMMA waste.^15−18^ However, such a high temperature not only demands high energy input but also increases the probability of forming highly reactive radical intermediates, leading to undesirable side reactions and impure MMA.^19−22^ The impurities compromise the quality of materials produced from subsequent repolymerization, posing a significant challenge for sustainable recycling practices.

**Scheme 1:**
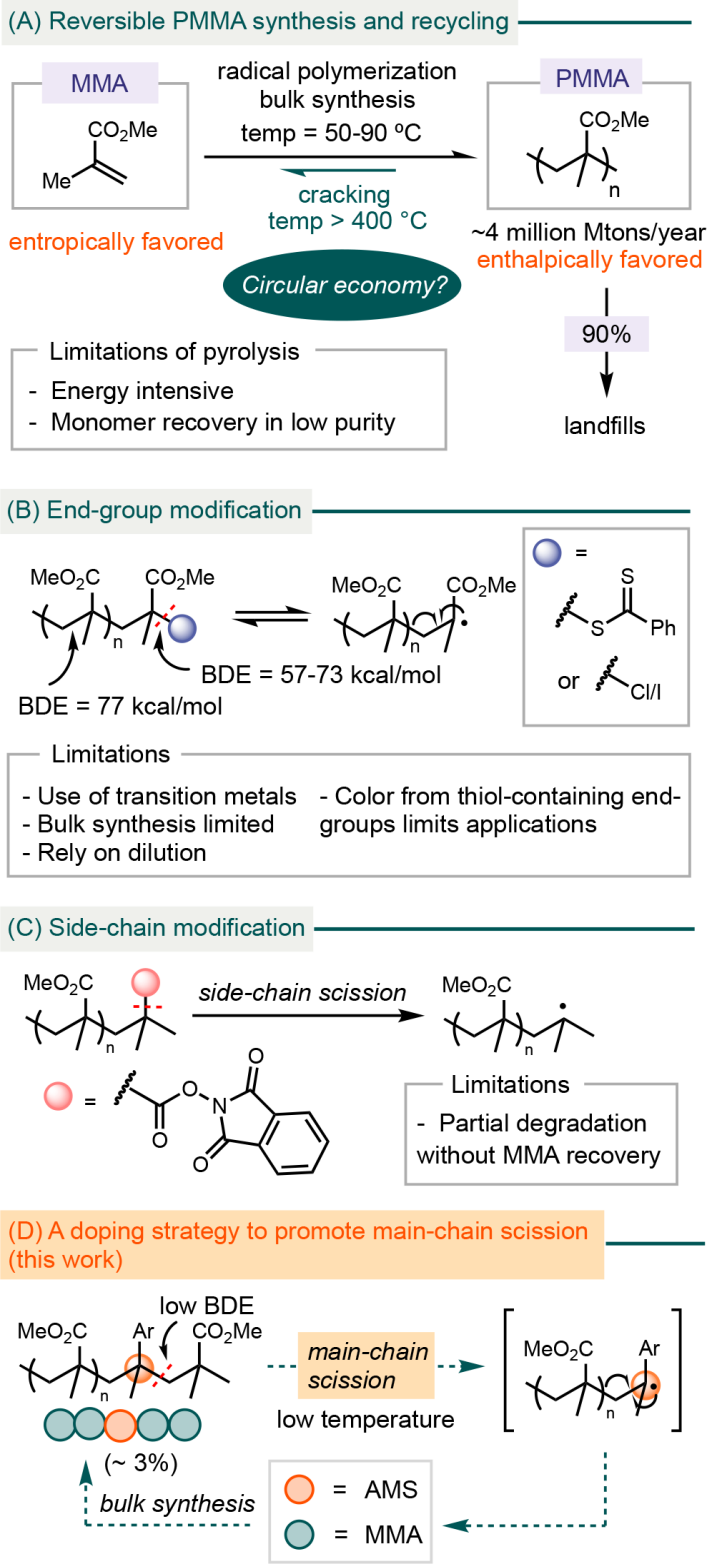
PMMA Depolymerization and the Proposed “Doping” Strategy

Recent advancements in plastic recycling have aimed to reduce the temperature required for PMMA depolymerization by incorporating functional groups with low bond dissociation energies (BDEs). PMMA prepared via atom transfer radical polymerization (ATRP)^23−27^ and reversible addition–fragmentation chain transfer (RAFT) polymerization^28−32^ retains chain-end functionalities that enable reversion to MMA under thermal and photoredox conditions (Scheme 1B). However, some of these methods necessitate the use of transition metal catalysts or expensive photocatalysts. Additionally, the thiocarbonate end-groups characteristic of RAFT polymers can cause undesirable coloration to the bulk material, limiting its use in applications where optical transparency is essential. Although ATRP and RAFT provide precise control over the molecular weight and polydispersity, they are not commonly employed in the production of bulk and commercial materials.

Alternative approaches to PMMA degradation involve copolymerizing MMA with redox-active^33,34^ or halogen-containing monomers.^35,36^ These copolymers can undergo side chain scission upon photoinduced or transition-metal-catalyzed single-electron transfer (SET), resulting in lower molecular weight oligomers (Scheme 1C). However, these methods have not yet demonstrated the capability to recover the monomer, a critical step for achieving a closed-loop recycling process for PMMA.

We hypothesize that “doping” PMMA with a small amount of a comonomer could introduce a weak bond within the carbon–carbon backbone. This modification would enable main-chain scission of the resulting polymer at moderate temperatures (Scheme 1D). Such a strategy has been utilized to improve polymerization and depolymerization kinetics of polyesters,^37^ but has not yet been applied to PMMA. Poly(α-methylstyrene) exhibits a low ceiling temperature (T_c_) of 66 °C, at which the rates of polymerization and depolymerization become equal. The low ceiling temperature stems from the stability of tertiary benzyl radicals.^38,39^ In this report, we demonstrate that doping PMMA with just 3% of α-methylstyrene (AMS) analogues allows for the polymer to thermally revert to MMA efficiently and selectively under mild conditions, while maintaining the valued properties of PMMA. Notably, our method is suitable for bulk synthesis and does not depend on precious metals or specific end-groups.

Our studies began with synthesizing PMMA doped with various AMS comonomers, carried out under bulk radical polymerization conditions at 85 °C (Figure 1). Ideally, the feed ratio of AMS should correlate with its incorporation into PMMA, without negatively affecting the mechanical strength and the optical clarity of PMMA. When MMA **1** was copolymerized with 4 mol % of AMS comonomers, the resulting P(MMA-*co*-AMS) **2**–**7** consistently exhibited approximately 3 mol % incorporation of AMS (Figure 1A).

**Figure 1:**
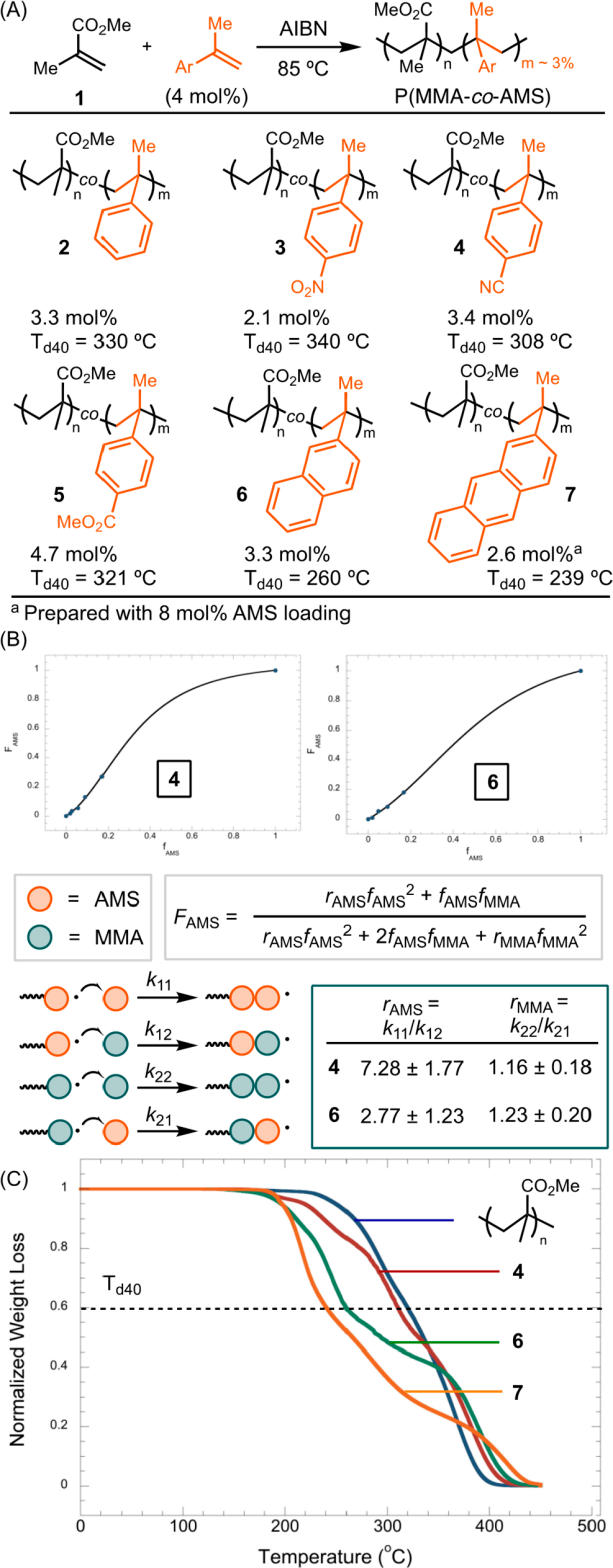
(A) Copolymerization of MMA with AMS derivatives. Conditions: MMA (2.00 mmol), AMS (4 mol %), AIBN (AIBN = azobis(isobutyronitrile)) (1.3 mol %), 85 °C. (B) Mayo–Lewis plots for P(MMA-*co*-AMS) **4** and **6**. (C) TGA of PMMA and P(MMA-*co*-AMS) **4**, **6**, and **7** under a nitrogen atmosphere.

We determined the relative reactivity ratios of AMS (r_AMS_) to MMA (r_MMA_) for P(MMA-*co*-AMS) **4** and **6** by conducting a series of bulk copolymerization reactions in which the starting AMS mole fraction (f_AMS_ = [M_AMS_]/[M_AMS+MMA_], f_AMS_ + f_MMA_ = 1) was varied. We then performed ^1^H NMR analysis of the resulting copolymers to determine the mole fraction of AMS incorporated into the copolymer (F_AMS_ = d[M_AMS_]/d[M_AMS+MMA_]) (Figure 1B). Fitting the data according to the Mayo–Lewis model^40^ indicates that an AMS radical tends to add to another molecule of AMS (r_AMS_ > 1), while an MMA radical shows nearly equal reactivity toward both AMS and MMA (r_MMA_ ≈ 1). In the presence of a large excess of MMA, the polymerization conditions for preparing P(MMA-*co*-AMS) likely lead to random incorporation of AMS (cf. Figure S2).

Copolymers **4**, **5**, and **6** exhibited average molecular weights (M_w_) on the order of 10^6^–10^7^ Da, consistent with that of commercial high-quality PMMA,^41^ whereas **7** displayed an average M_w_ on the order of 10^4^ Da (Table S1).^42^ Differential scanning calorimetry (DSC) experiments determined that the glass transition temperatures (T_g_) of **4**, **6**, and **7** range from 104 to 117 °C, comparable to that of the PMMA homopolymer (Figures S45–S48).^43^

Thermogravimetric analysis (TGA) of **4**, **6**, and **7** indicated that P(MMA-*co*-AMS) began to decompose at lower temperatures compared to the homopolymer PMMA (T_d40_ = 320 °C) (Figure 1C); T_d40_ represents the temperature at which 40% of the polymer has degraded.^44^ P(MMA-*co*-AMS) **7** exhibited a significantly lower T_d40_ value of 239 °C.

Subsequently, we explored the thermostability of P(MMA-*co*-AMS). In solution, **4**, **6**, and **7** displayed no degradation after heating at 100 °C for 1 h and only 1% of MMA after 4 h (Figures S17–S19). In bulk, GPC analysis showed minimal decomposition of **4**, **6**, and **7** after bulk heating at 100 °C for 4 h (S20–S22). After a brief heating at 200 °C for two min, **4** and **6** showed insignificant degradation, while **7** displayed minor decomposition. Moreover, isothermal TGA experiments revealed no weight loss at 100 °C (Figures S24–S26). These results suggest that P(MMA-*co*-AMS) is thermally stable under service conditions of PMMA.

We investigated the depolymerization of P(MMA-*co*-AMS) in solution at 150 °C—a temperature 250 K below conventional pyrolysis temperatures (Figure 2A). While the PMMA homopolymer underwent no conversion, heating a solution of P(MMA-*co*-AMS) in C_6_D_6_ led to various degrees of reversion to MMA. We added 4-methoxyphenol (MEHQ) to suppress the repolymerization of MMA during the cooling phase following depolymerization. The efficiency of depolymerization did not display a direct correlation to either the electronic effect of AMS or its T_d40_ value. P(MMA-*co*-AMS) **6** and **7** both contain extended conjugation in the AMS moiety and exhibit similar TGA profiles. However, when heating to 150 °C, **7** afforded a high yield of MMA at 80%, whereas the same protocol with **6** resulted in only a 23% yield of MMA. Adding 3 equiv of TEMPO inhibited the reaction, suggesting that depolymerization is mediated by radical intermediates.

**Figure 2:**
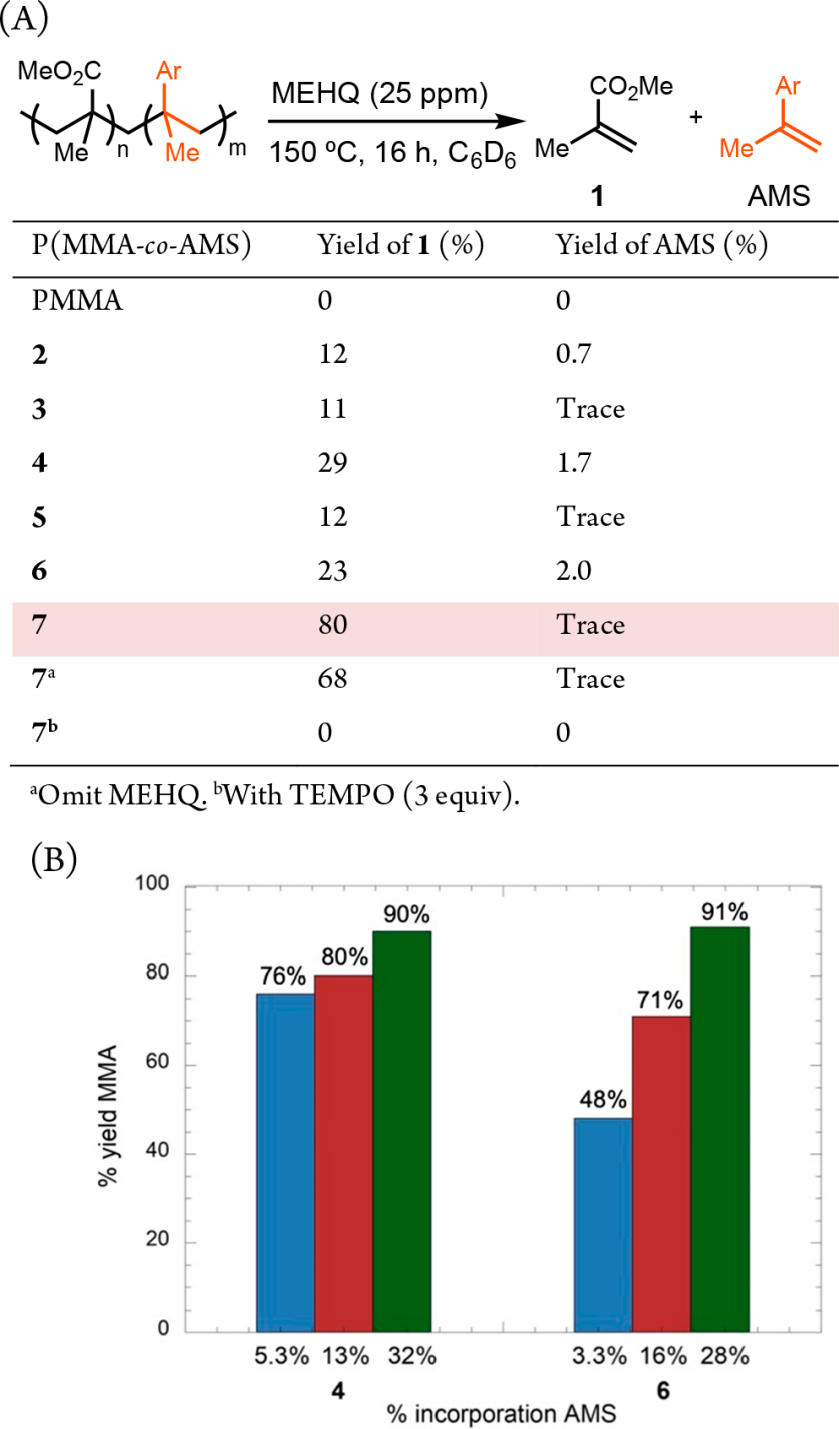
Depolymerization of P(MMA-*co*-AMS) copolymers (A) and effect of mol % of AMS comonomer (B). Conditions: polymers (1 mg), MEHQ (25 ppm), 200 °C, and C_6_D_6_ (0.7 mL). .

We evaluated the depolymerization conversion as a function of the percentage of AMS incorporation for P(MMA-*co*-AMS) **4** and **6** (Figure 2B). Increasing the percentage of AMS incorporation marginally affected the T_d40_ values (Figures S30 and S31). However, both copolymers **4** and **6** exhibited higher conversion to MMA with a higher AMS mol %. Notably, with approximately 30% AMS, the yield of MMA increased to 90%.

To validate the viability of AMS-doped PMMA for monomer recovery through catalyst-free depolymerization, we conducted bulk depolymerizations of P(MMA-*co*-AMS) **4**, **6**, and **7**, and a mixture of plastic wastes (Scheme 2). Depolymerization of **4** and **6** afforded **1** in 46% and 53% yields, respectively. The depolymerization of **7** at 180 °C resulted in the recovery of **1** in 76% yield. Additionally, depolymerization of **6** amidst various plastics, including polypropylene (PP), polyethylene (PE), polyethylene terephthalate (PET), and polystyrene (PS), selectively produced **1** in high purity. Furthermore, we successfully demonstrated that the recovered MMA from the depolymerization of **4** could be repolymerized without further purification (Figure S16).

**Scheme 2:**
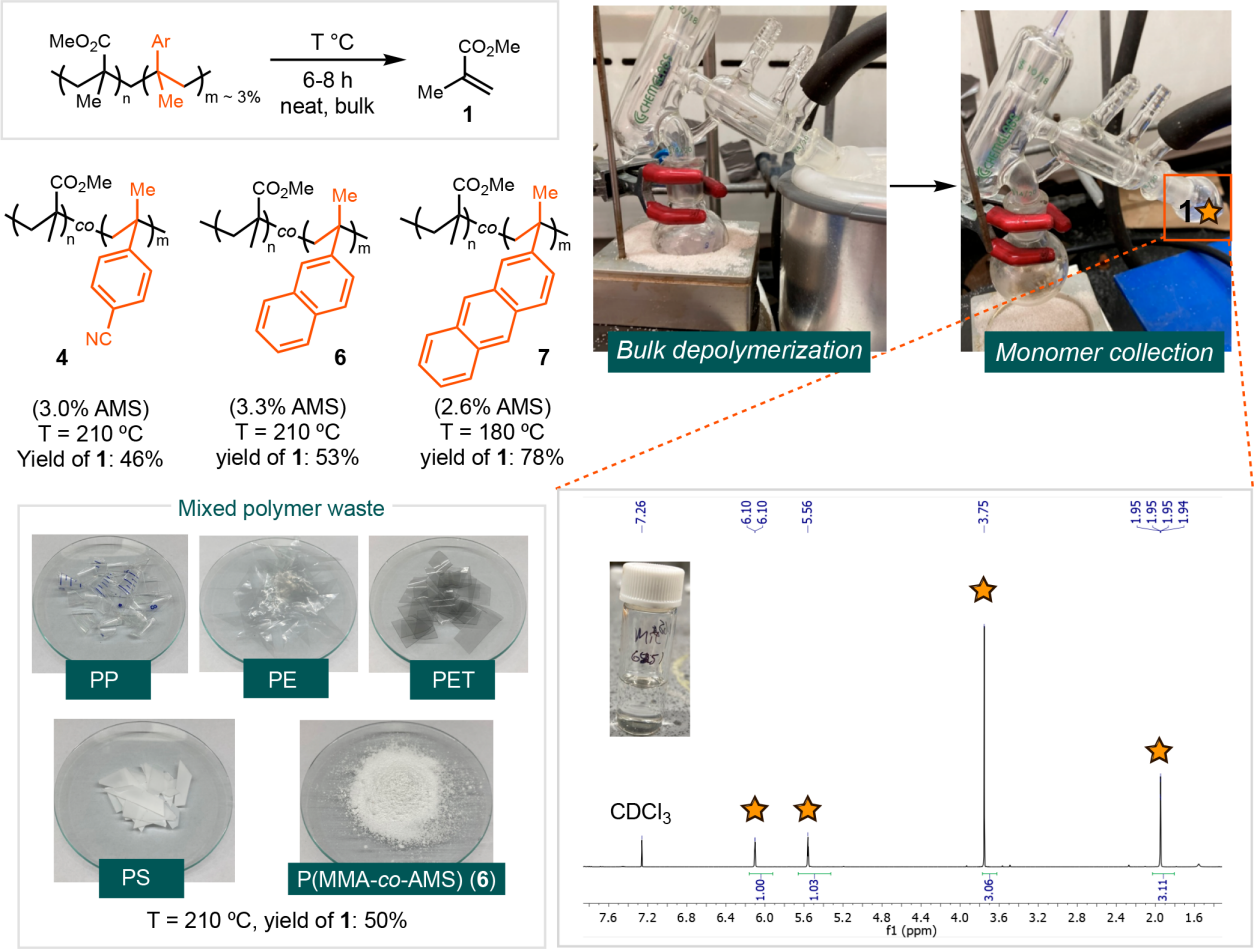
Bulk Depolymerization of P(MMA-*co*-AMS)

We prepared solvent-cast polymer films of **4**, **6**, and **7** for visual comparison with a PMMA homopolymer and a RAFT polymer **8**.^30^ P(MMA-*co*-AMS) films are optically transparent and colorless, indistinguishable from the PMMA homopolymer (Figure 3A). The UV–vis transmittance spectra of **4** and **6** revealed no notable deviation from the PMMA homopolymer in the region above 300 nm (Figure 3B). Meanwhile, **7** exhibited significant UV absorption below 390 nm, but it maintained excellent transmittance in the visible light region above 400 nm. This transmittance aligns with the high transparency observed in the films. Notably, the UV absorption of **7** suggests potential applications in sunscreen devices.

**Figure 3:**
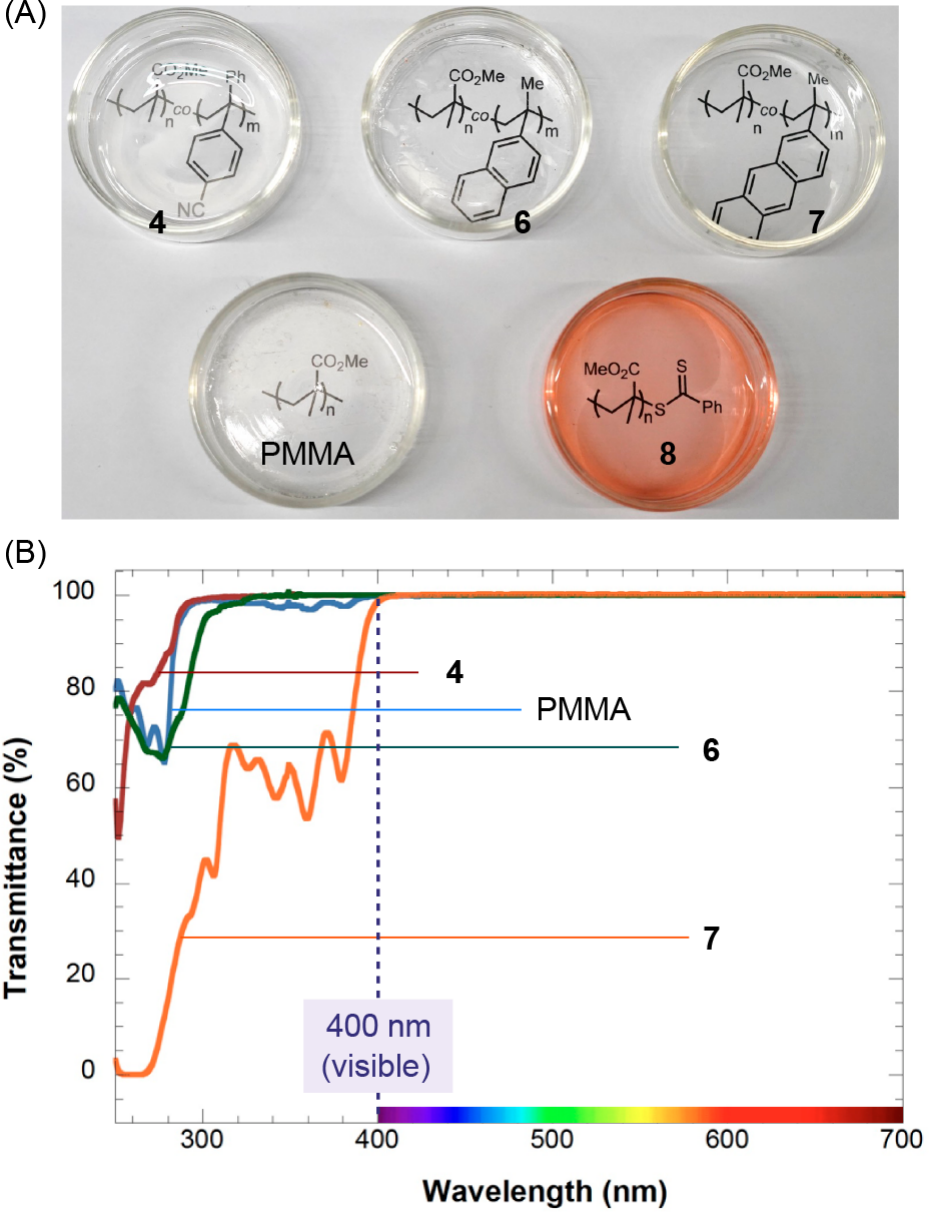
(A) Solvent-cast films of P(MMA-*co*-AMS) copolymers exhibit colorless transparency. (B) UV–vis transmittance spectra of PMMA and P(MMA-*co*-AMS) copolymers.

Furthermore, we examined the tensile strength of a thin film of **6** (Figure S33). The Young’s modulus of **6** was 798 MPa with an elongation at break of 2.5%, comparable to that of PMMA at 626 MPa. Therefore, copolymerization with AMS did not compromise the optical and mechanical properties of PMMA.

The excellent depolymerization efficiency of doped PMMA in depolymerization at low temperatures prompted us to investigate the mechanistic rationale. We employed density functional theory (DFT) calculations on model compounds **9**, **10**, and **11**, using Gaussian 16 and applying the M06-2X functional^45^ with the def2-TZVP basis set (Scheme 3A).^46^ Our results suggest that the carbon–carbon bonds between MMA and AMS in **10** and **11** are significantly weaker compared to the carbon–carbon bonds in the backbone of **9**. Monitoring the depolymerization with ^1^H NMR spectroscopy demonstrated rapid sharpening of the aromatic peaks, suggesting formation of an AMS monomer (Figures S9–S11). Additionally, the concentration of AMS reached stead-state after an hour.

**Scheme 3:**
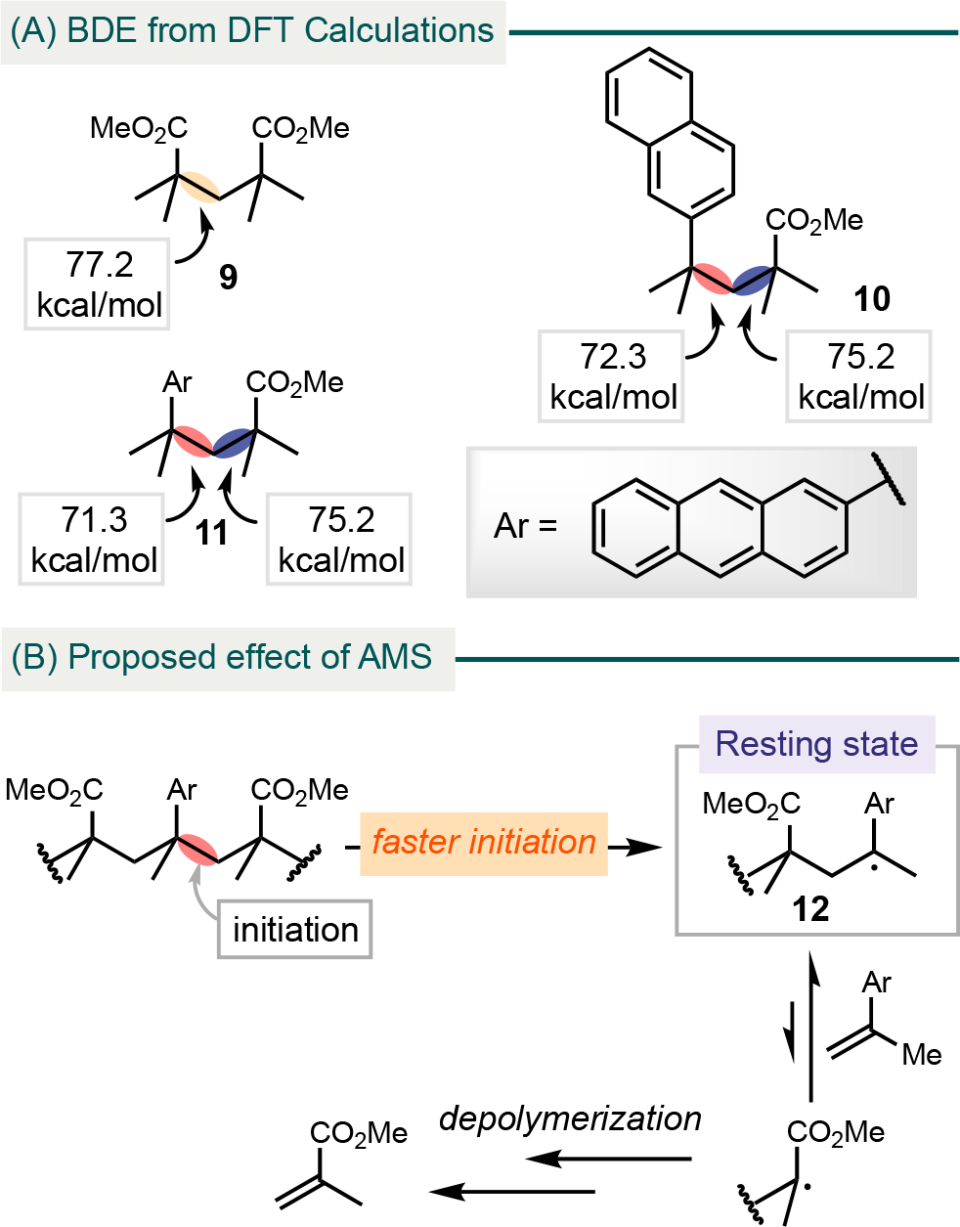
DFT Calculations of Model Compounds (A) and Mechanistic Rationale for the Depolymerization of P(MMA-*co*-AMS) (B)

Data above provide a mechanistic account for the facile depolymerization of AMS-doped PMMA (Scheme 3B). The presence of weak bonds in the polymer backbone and the rapid formation of AMS monomer suggests that depolymerization is initiated by random scission of linkages involving AMS.^47^ Furthermore, AMS can facilitate depolymerization by reversibly forming stable tertiary benzyl radical intermediate **12** during depolymerization, which serves as a resting state that modulates the reactivity and prevents undesired side pathways. The different depolymerization efficiency among various AMS analogues may be attributed to the steric effect, where bulkier AMS leads to more favorable elimination.

In conclusion, we have developed a “doping” strategy by integrating a small percentage of an AMS analogue into PMMA copolymers. This approach preserves the mechanical and optical properties of PMMA, while enabling facile depolymerization at low temperatures. Even when PMMA is mixed with other plastics, this method allows for the recovery of MMA in high yields and purity. This technique paves the way for substituting traditional commodity materials with those that can be easily reverted to their monomeric forms, making a significant step toward a circular economy.

## Supporting Information Available

Detailed experimental procedures, characterization data, and details of DFT calculations (PDF)
