# Supplementary material for: Implementing a Doping Approach for Poly(methyl methacrylate) Recycling in a Circular Economy
Source: J Am Chem Soc. 2024 Feb 21;146(9):5786–92. doi: 10.1021/jacs.3c13223 (PMC10921398; doi:10.1021/jacs.3c13223)
Supplement: Supplementary file 1 — ja3c13223_si_001.pdf [file ja3c13223_si_001.pdf]

Supporting Information

Implementing a Doping Approach for Poly(Methyl Methacrylate) (PMMA) Recycling in a Circular Economy

Mason T. Chin,[1] Tiangang Yang,[2] Kevin P. Quirion,[3] Christina Lian,[1] Peng Liu,[3*] Jie He,[2*] and Tianning Diao[1*]

[1]Department of Chemistry, New York University, 100 Washington Square East, New York, NY 10003, United States

[2]Department of Chemistry, University of Connecticut, Storrs, CT 06269, United States

[3]Department of Chemistry, University of Pittsburgh, Pittsburgh, PA 15260, United States

E-mail: diao@nyu.edu; jie.he@uconn.edu; pengliu@pitt.edu

This PDF file includes: **Page**

1. General Experimental Details....1
2. Synthesis of α-Methylstyrene Comonomers....3
3. Procedure for Bulk Polymerization of MMA with Comonomer....4
4. Discussion of Triad Sequences in P(MMA-*co*-AMS) Copolymers....5
5. Procedure for the Depolymerization of PMMA Copolymers....6
6. Time Course of Solution-Phase Depolymerization....9
7. Bulk Depolymerization of P(MMA-*co*-AMS) Copolymers....11
8. Stability Tests of P(MMA-*co*-AMS) Copolymers....15
9. TGA Thermograms....19
10. Stress-Strain Curve....24
11. NMR Spectra....25
12. DSC Spectra....30
13. DFT Calculations....32
14. Cited References....41

1. General Experimental Details

Equipment and Methods

All air- and moisture- sensitive manipulations were performed using Schlenk technique. Chemical shifts of the ^{1}H resonances are reported in ppm relative to tetramethylsilane, with the residual solvent resonance ($CDCl_3$, δ = 7.26 ppm, acetone-d_6, δ = 2.05 ppm, C_6D_6, δ = 7.16 ppm)

as the internal reference. Spectra are reported as the following: chemical shift (δ ppm), multiplicity (s = singlet, b s = broad singlet, d = doublet, t = triplet, q = quartet, m = multiplet), coupling constant (Hz), and integration. Chemical shifts of the ^{13}C were reported in ppm relative to tetramethylsilane with the solvent resonance used as the internal reference ($CDCl_3$, δ = 77.2 ppm acetone-d_6, δ = 29.84 ppm). High resolution mass spectra (HRMS) were collected on an Agilent 6224 TOF LC/MS. UV-Vis spectra were obtained using a Cary 100 UV-Visible Spectrophotometer. Thermogravimetric analysis (TGA) was performed using a TGA Q500-1732 in a N_2 atmosphere. Samples were annealed from 25 to 600 °C with a rate of heating of 2 or 10 °C/min under N_2 flow. Differential scanning calorimetry (DSC) was performed using a TA Instruments DSC Q20. Experiments were carried out under N_2 flow with a heat ramp of 10 °C/min from -50 °C to 200 °C. Gel permeation chromatography (GPC) was conducted on a Waters GPC-1 (Waters 1515 HPLC pump & Waters 717 Autoinjector) equipped with Waters 2414 Refractive Index Detector (RI) and three Jordi Gel fluorinated DVB columns (1-100K, 2-10K & 1-500A). Tetrahydrofuran (THF) was used as an elution solvent and polystyrene as the standard for calibration of the molecular weight and dispersity. Mechanical tests were carried out on films using a TA-XT plus C texture analyzer (Stable Micro Systems) equipped with a 50 kg load cell at a speed of 2 mm/s and the stress values were calculated by Exponent software (Stable Micro systems, UK). All the copolymer samples were prepared as thin films and cut into rectangular shapes with a size of ~ 30 × 10 × 0.6 mm for data acquisition. Reactions were monitored by thin-layer chromatography (TLC) on Merck TLC silica gel 60 F254 plates and compounds were visualized by UV light (254 nm) or staining with $KMnO_4$.

Reagents and Solvents

All commercially available compounds were purchased and used as received, unless otherwise noted. Methyl methacrylate (MMA) was passed through a plug of basic alumina to remove inhibitor before use. THF, MeOH, EtOH, diethyl ether, and toluene were dried and deoxygenated by passing through alumina in a solvent purification system. Dimethyl sulfoxide (DMSO) was dried over CaH_2, distilled, and degassed before use. C_6D_6 was stored over molecular sieves for several weeks before use.

2. Synthesis of α-methyl- styrene comonomers

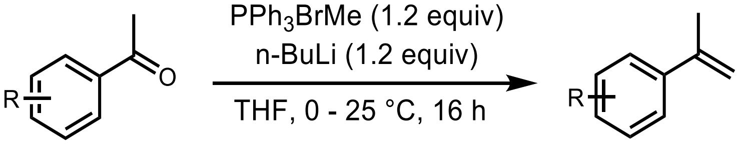

In a flame-dried round bottom flask, PPh_3BrMe (1.2 equiv) was added, and the flask was purged with N_2. THF (0.5 M) was added to yield a white slurry. The mixture was cooled to 0 °C in an ice bath and n-BuLi (1.2 equiv, 2.5 M) was added dropwise over 5 minutes. The reaction stirred for 1 hour at 0 °C. A solution of the ketone (1 equiv) in THF (1 M) was added dropwise and the reaction stirred overnight at room temperature. After overnight stirring, the reaction is quenched with sat. aq. NH_4Cl and extracted with EtOAc 3x. The organic layers were combined, dried over Na_2SO_4, filtered and concentrated. The crude residue was purified *via* column chromatography to yield the α-methyl styrene product.

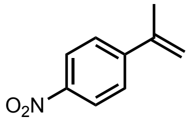

1-nitro-4-(prop-1-en-2-yl)benzene (SI-1).[1] Purified with 30:1 hexanes:EtOAc. 37% yield. ^1H NMR (500 MHz, $CDCl_3$) δ 8.19 (d, J = 9.0 Hz, 2H), 7.59 (d, J = 9.0 Hz, 2H), 5.52 (m, 1H), 5.29 (m, 1H), 2.19 (s, 3H).

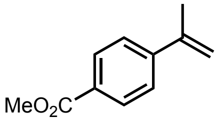

Methyl 4-(prop-1-en-2-yl)benzoate (SI-2).[2] Purified with 10:1 hexanes:EtOAc. 67% yield. ^1H NMR (400 MHz, $CDCl_3$) δ 8.02 (d, J = 8.5 Hz, 2H), 7.54 (d, J = 8.5 Hz, 2H), 5.49 (m, 1H), 5.22 (m, 1H), 3.94 (s, 3H), 2.20 (s, 3H).

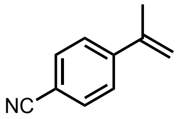

4-(prop-1-en-2-yl)benzonitrile (SI-3).[3] Purified with 20:1 hexanes:EtOAc. 20% yield. ^1H NMR (500 MHz, $CDCl_3$) δ 7.64 (d, J = 8.5 Hz, 2H), 7.57 (d, J = 8.5 Hz, 2H), 5.49 (m, 1H), 5.27 (m, 1H), 2.18 (s, 3H).

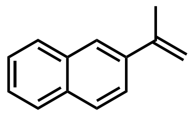

2-(prop-1-en-2-yl)naphthalene (SI-4).[4] Purified with hexanes. 90% yield. ^{1}H NMR (400 MHz, $CDCl_3$) δ 7.86 – 7.78 (m, 4H), 7.70 – 7.65 (m, 1H), 7.50 – 7.42 (m, 2H), 5.53 (m, 1H), 5.20 (m, 1H), 2.28 (s, 3H).

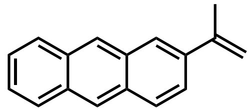

2-(prop-1-en-2-yl)anthracene (SI-5).[4] Purified with hexanes. 88% yield. ^{1}H NMR (400 MHz, $CDCl_3$) δ 8.40 (d, 2H), 8.02 – 7.92 (m, 4H), 7.70 (m, 1H), 7.49 – 7.43 (m, 2H), 5.62 (s, 1H), 5.26 (s, 1H), 2.32 (s, 3H).

3. Procedure for Bulk Polymerization of MMA with Comonomer

In a 4 mL vial with a septum cap, AIBN (0.013 equiv) and comonomer (if solid, 0.04 equiv) was combined, and the system was purged with N_2 (for polymer **7**, 0.04 equiv AIBN and 0.08 equiv of comonomer was used). MMA (0.2 mmol) and comonomer (if liquid, 0.04 equiv) was added *via* syringe and the reaction was heated at 85 °C for 16 h. After allowing the reaction to cool to room temperature, the resulting mixture was dissolved in DCM until complete dissolution. The resulting solution was diluted in MeOH to precipitate a white solid. The mixture was centrifuged, and the supernatant was discarded. This process was repeated 2x to obtain the purified polymer as a solid material. After drying *in vacuo* for several hours, ^{1}H NMR were taken in acetone-d_6 and % incorporation of comonomer were determined by comparing the intensity of the aromatic signals of the comonomer with the –OMe peaks of the polymer.

Table SI-1. GPC Characterization of P(MMA-*co*-AMS) Copolymers

P(MMA-*co*-AMS)	Mn (Da)	Mw (Da)	Đ
**2**	6.83×10^4	1.00×10^5	1.47
**3**	1.07×10^4	1.61×10^4	1.51
**4**	3.58×10^5	2.31×10^6	6.45
**5**	2.70×10^6	1.57×10^7	5.80
**6**	1.20×10^6	7.85×10^6	6.55
**7**	5.75×10^3	1.99×10^4	3.47
PMMA	1.30×10^5	2.61×10^5	2.01

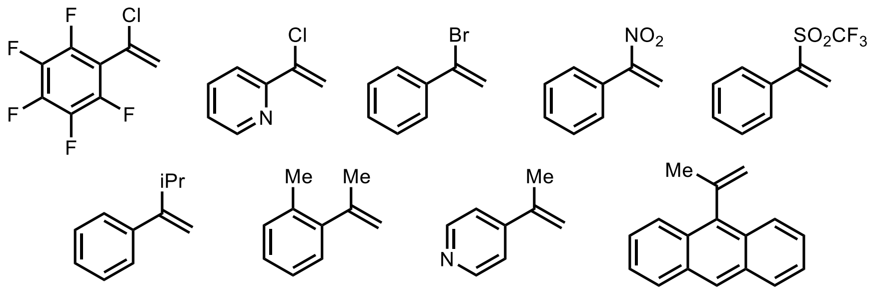

Figure S1. AMS derivatives failed to undergo copolymerization.

4. Discussion of Triad Sequences in P(MMA-*co*-AMS) copolymers

Examining the –OMe region of **4**, **6**, and **7** and comparing them to a PMMA homopolymer, we tentatively assigned triad sequences that could exist within our copolymers.[5] Given the low feed ratio of AMS to MMA and the high reactivity ratio of the AMS comonomers, we assigned the downfield most –OMe peaks to be a mixture of rrm and rmr. Likewise, we assigned the most upfield –OMe peaks to be a mixture of mmr and mrm and the large peak at 3.64 ppm to be mmm, which was determined *via* comparison to a PMMA homopolymer. These peaks also increase as the mole fraction of AMS incorporated into the copolymer increases. However, it is important to note that this assignment is primarily speculative, as we lack standards for direct comparison.

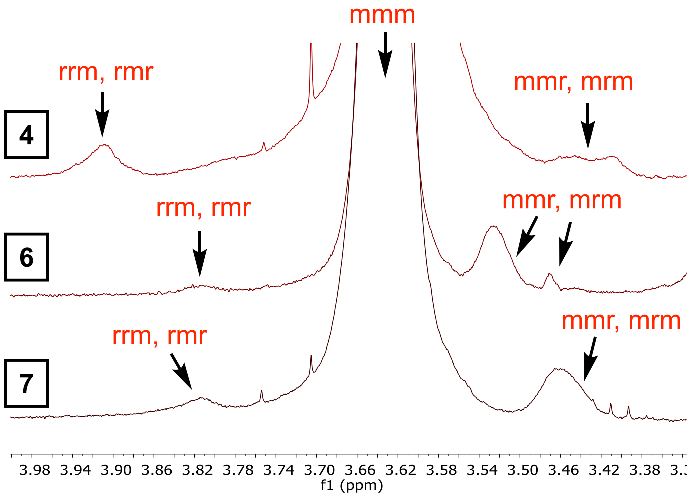

	mmm	mmr, mrm	rrm, rmr
**4**	95.4%	1.7%	2.8%
**6**	93.0%	5.7%	1.3%
**7**	96.2%	2.0%	1.8%

m = MMA
r = AMS

Figure S2. ^{1}H NMR analysis of **4**, **6**, and **7** and tentative assignments.

5. Procedure for the Depolymerization of P(MMA-*co*-AMS) copolymers

In an oven-dried J-young tube, 1 mg of polymer was dissolved in 0.7 mL C_6D_6 containing 25 ppm of MEHQ. The mixture was evacuated and backfilled with N_2 *via* 3x freeze-pump-thaw cycles. The reaction was heated at 150 °C for 16 h. After allowing the reaction to cool to room temperature, 1 µL of nitromethane was added as an internal standard and ^1H NMR spectrum were recorded. Depolymerization conversions were calculated by comparing the integrations of the vinyl peaks of MMA and the –OMe peak of the remaining polymer. To ensure accurate calculation, depolymerization conversion was also determined by comparing the MMA peaks to the internal standard peaks. The conversions determined from the two methods deviated by <5%.

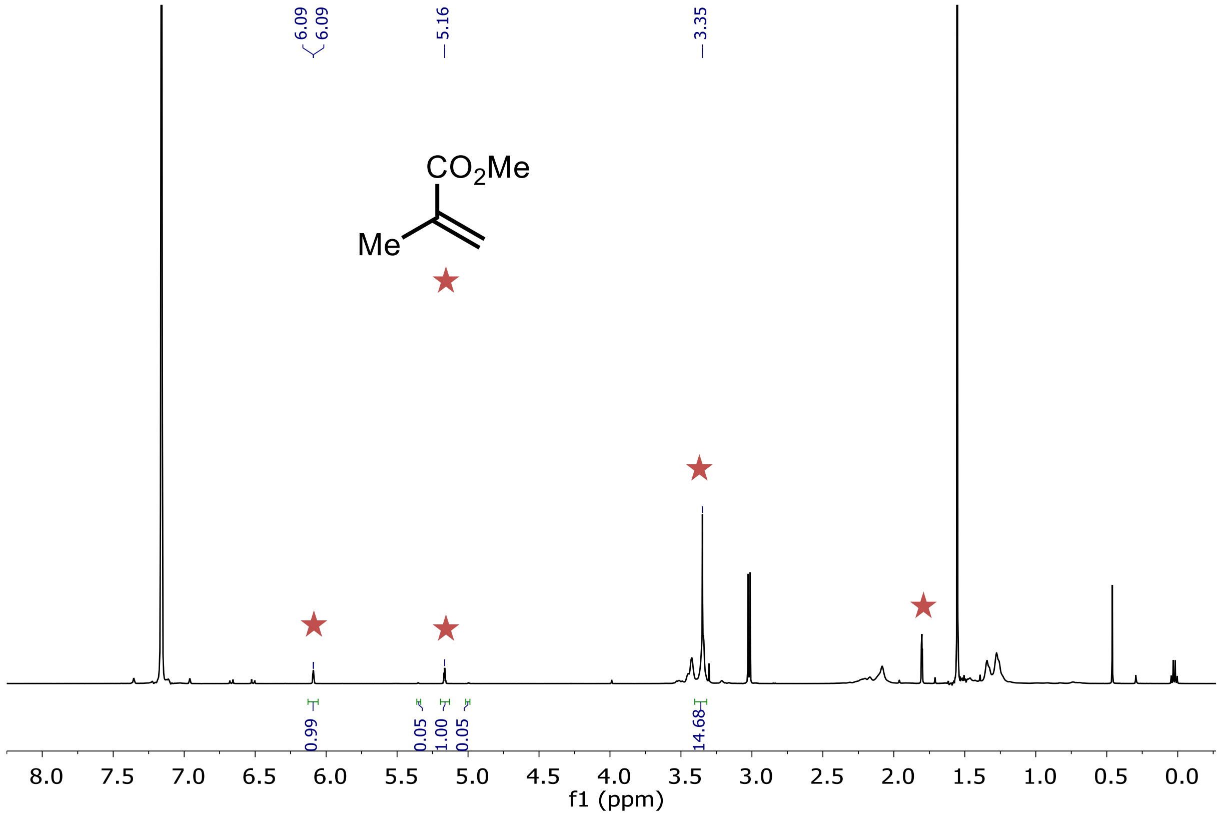

Figure S3. ^1H NMR spectrum of the depolymerization of **2** at 150 °C in a solution of C_6D_6.

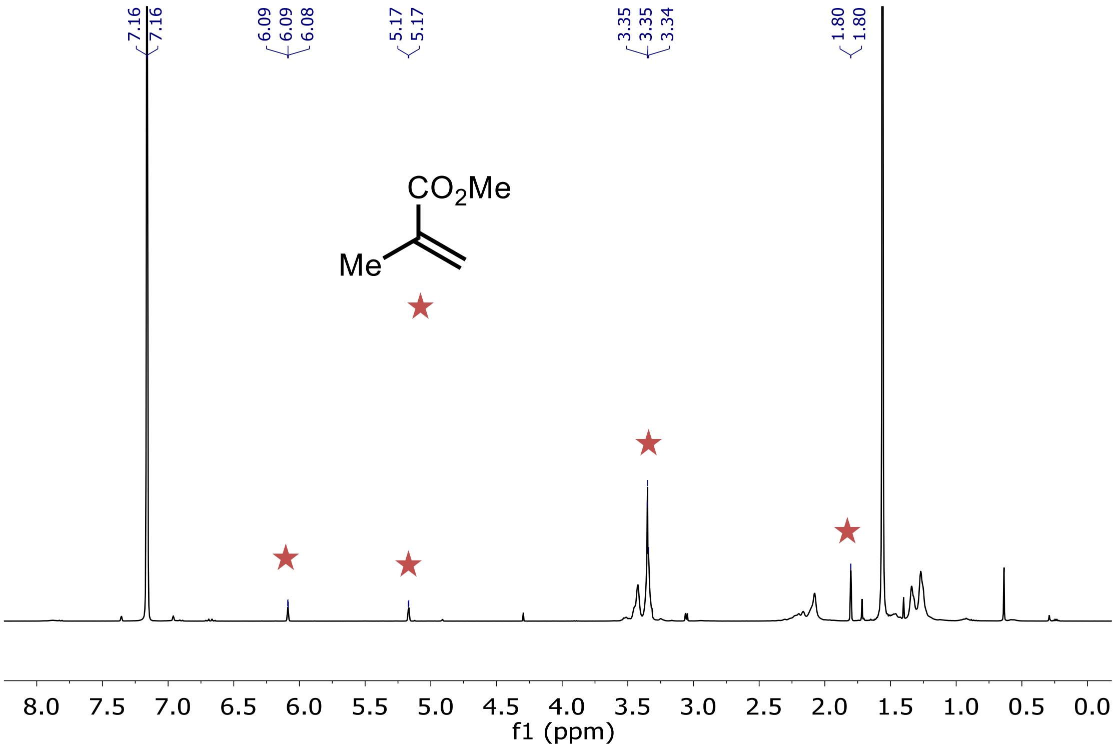

Figure S4. ^{1}H NMR spectrum of the depolymerization of at **3** at 150 °C in a solution of C_6D_6.

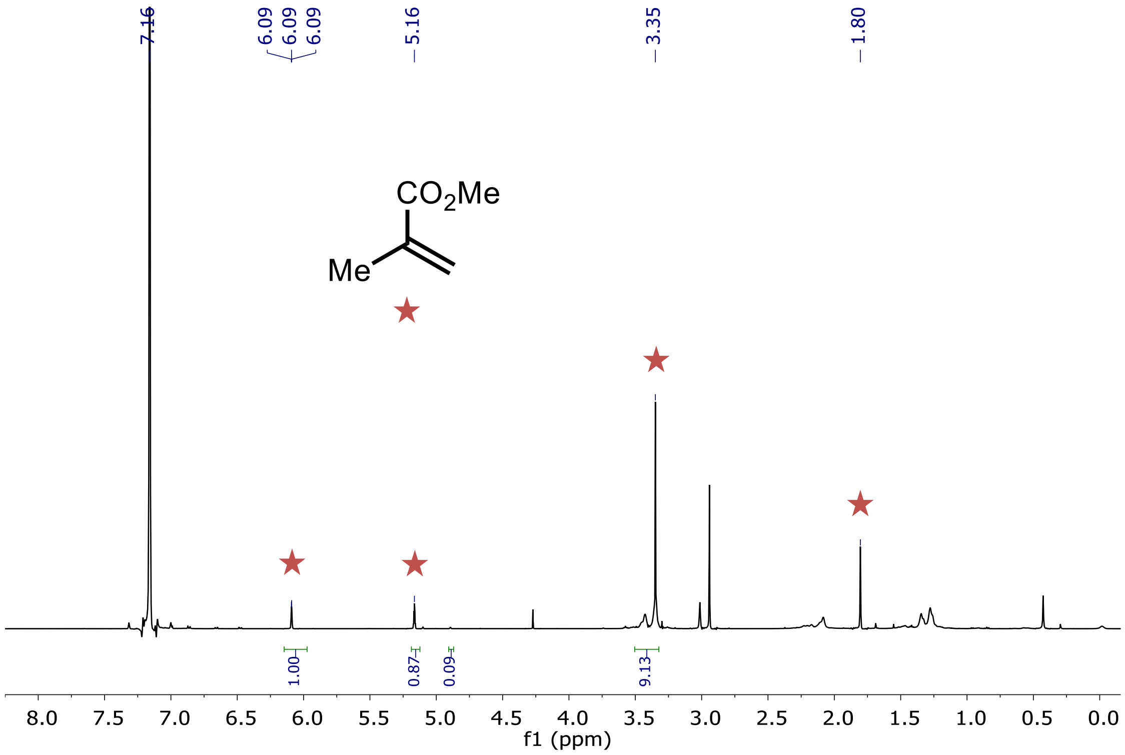

Figure S5. ^{1}H NMR spectrum of the depolymerization of **4** at 150 °C in a solution of C_6D_6.

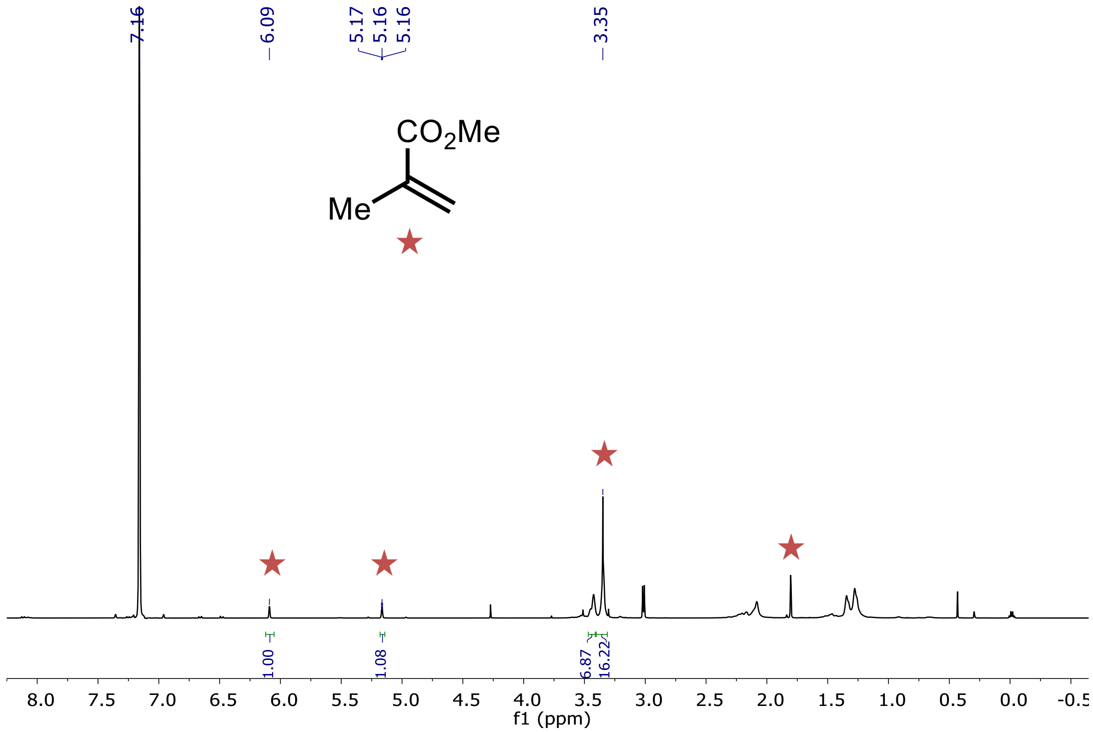

Figure S6. ^{1}H NMR spectrum of the depolymerization of **5** at 150 °C in a solution of C_6D_6.

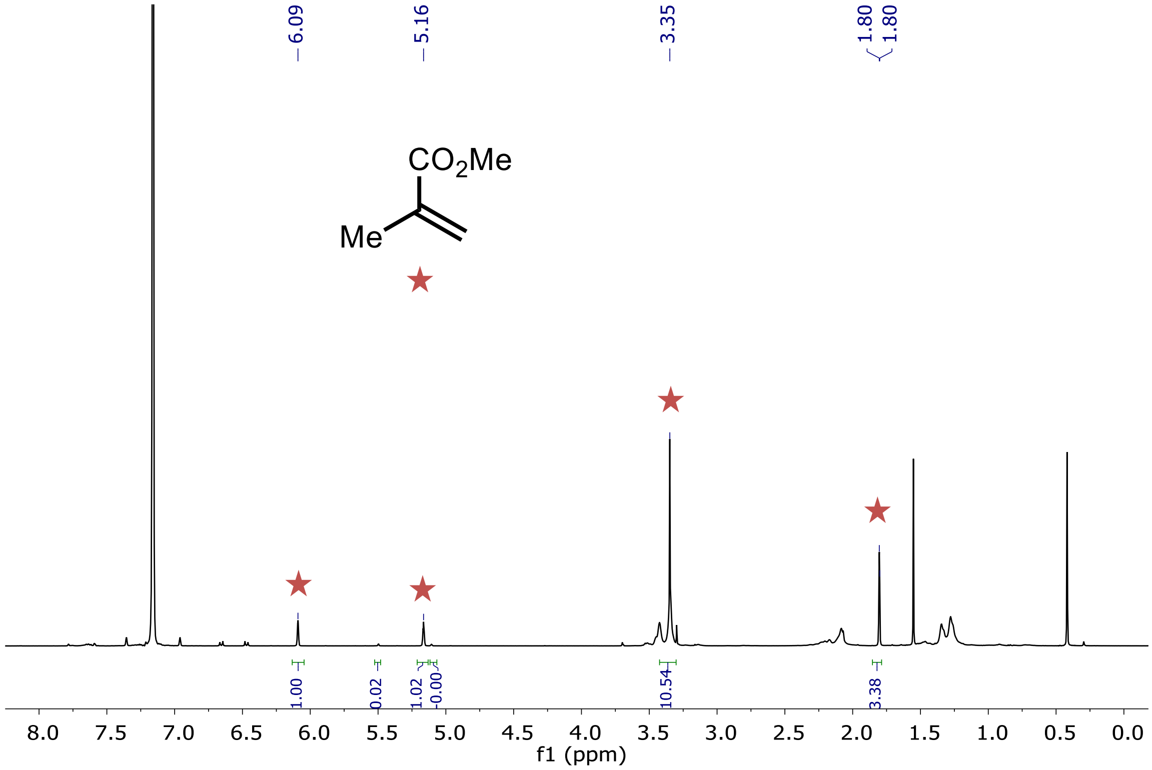

Figure S7. ^{1}H NMR spectrum of the depolymerization of **6** at 150 °C in a solution of C_6D_6.

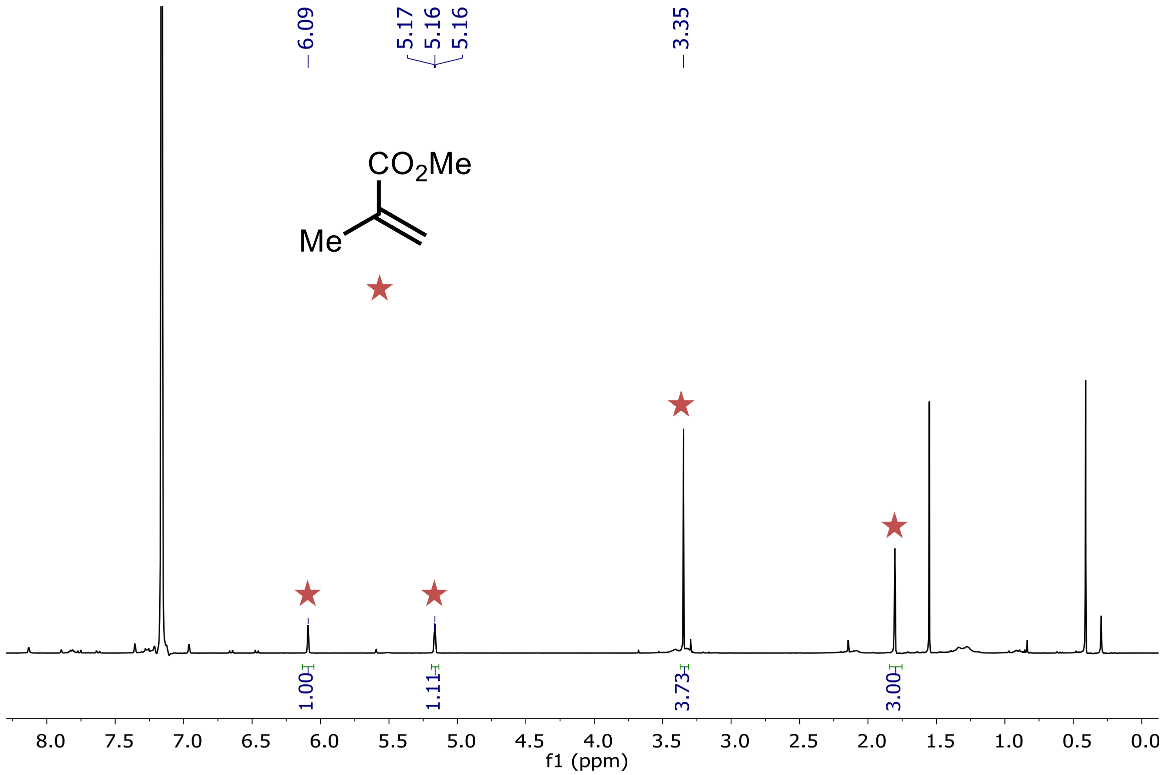

Figure S8. ^{1}H NMR spectrum of the depolymerization of **7** at 150 °C in a solution of C_6D_6.

6. Time Course of Solution-Phase Depolymerization

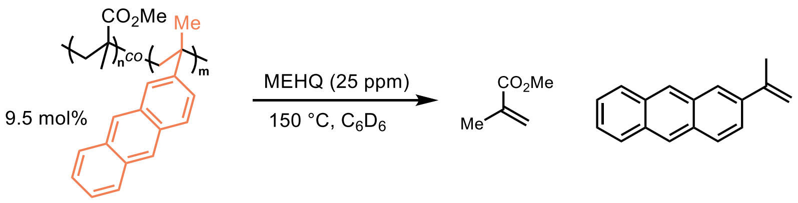

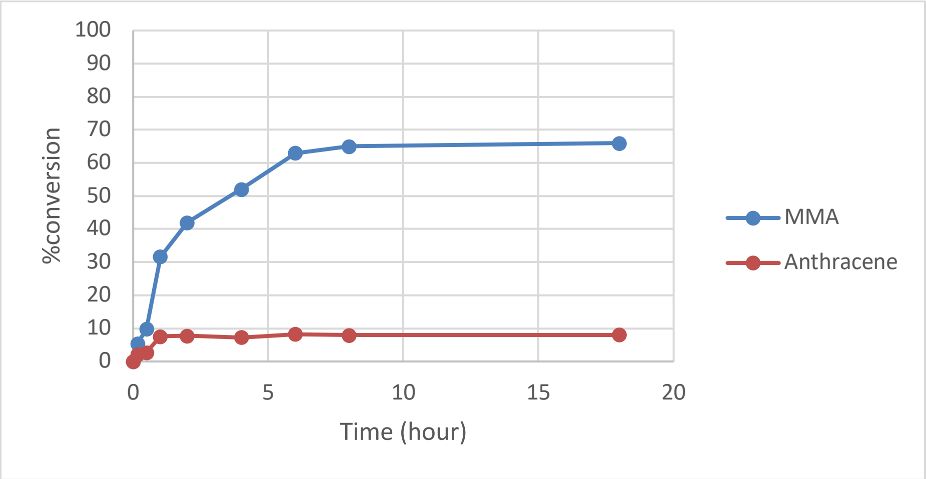

Figure S9. Time course for the depolymerization of P(MMA-*co*-AMS)$_{\text{anthracene}}$ at 150 °C in C_6D_6.

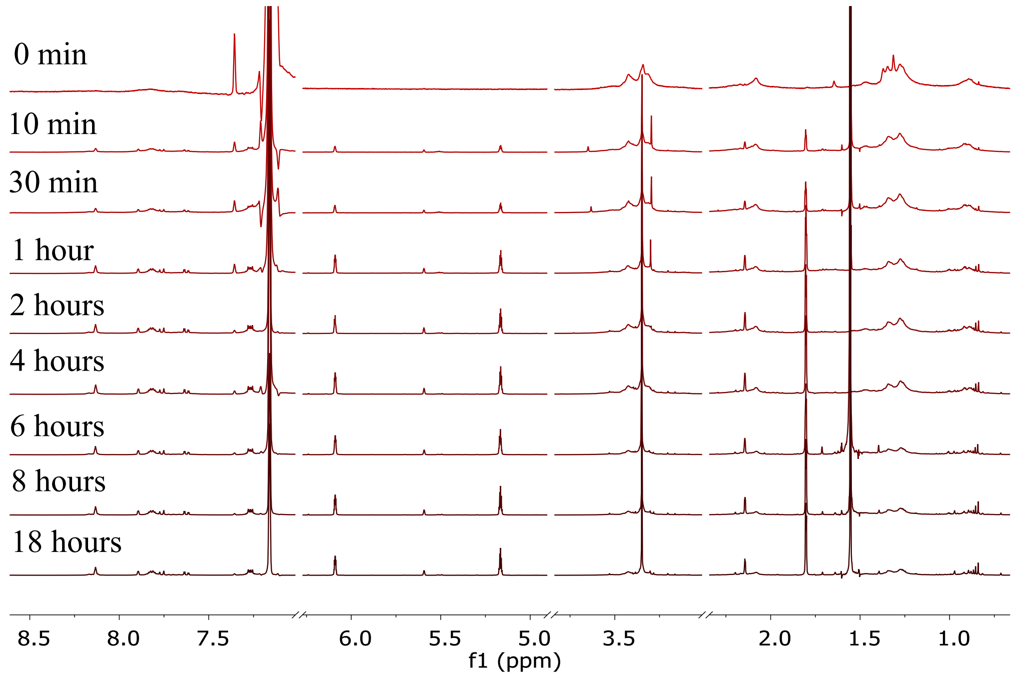

Figure S10. ^{1}H NMR spectra of a time course for the depolymerization of P(MMA-*co*-AMS)$_{anthracene}$.

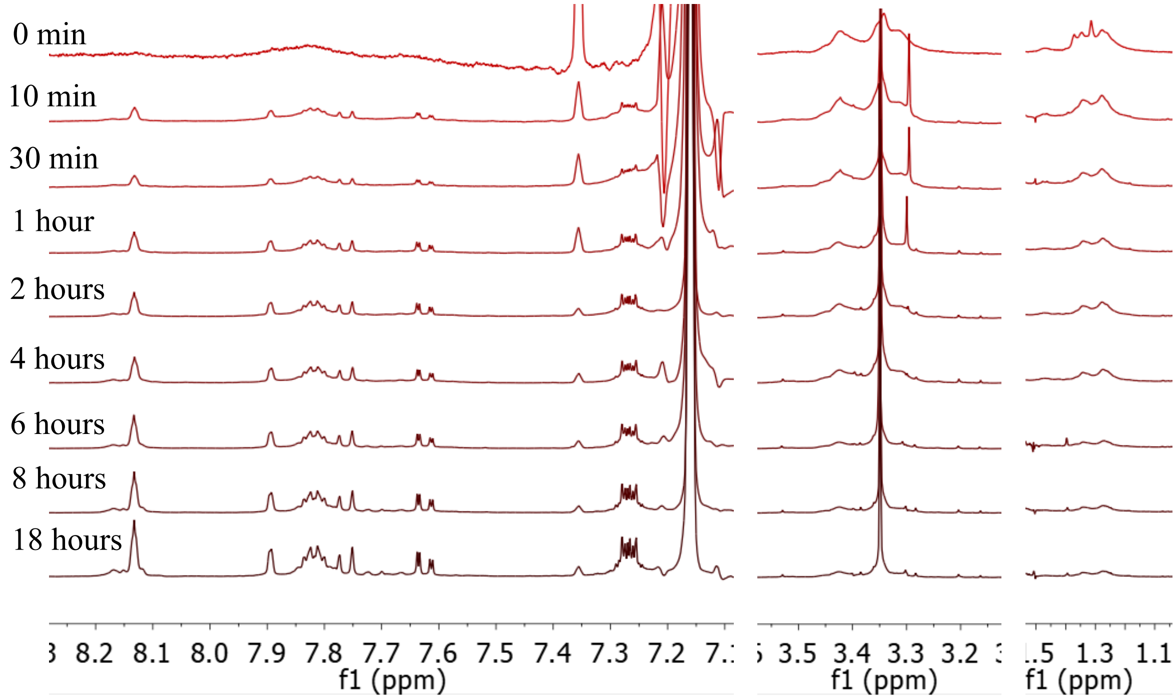

Figure S11. ^{1}H NMR spectra of a time course for the depolymerization of P(MMA-*co*-AMS)$_{anthracene}$ zooming in on polymer regions.

7. Bulk Depolymerization of P(MMA-*co*-AMS) copolymers

In a flame-dried round bottom flask, P(MMA-*co*-AMS) copolymer was added, and the flask was attached to a short path vacuum distillation head. Under vacuum, the reaction was heated for 6-8 h (**4** and **6** were performed for 8 hours, **7** and the reaction with mixed plastic waste were performed for 6 hours). After the reaction, the system was backfilled with N_2 and the distillate was analyzed by ^{1}H NMR.

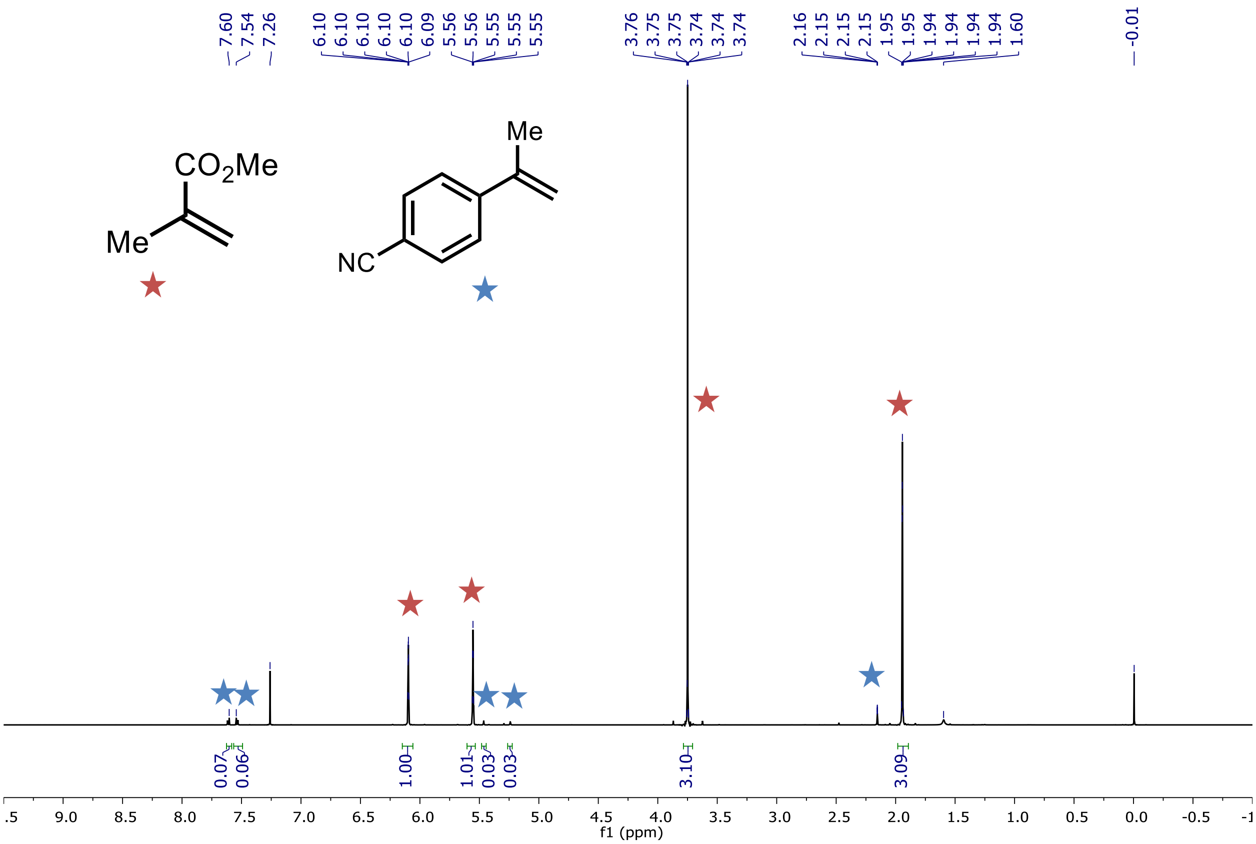

Figure S12. ^{1}H NMR spectrum of distillate after bulk depolymerization of **4**.

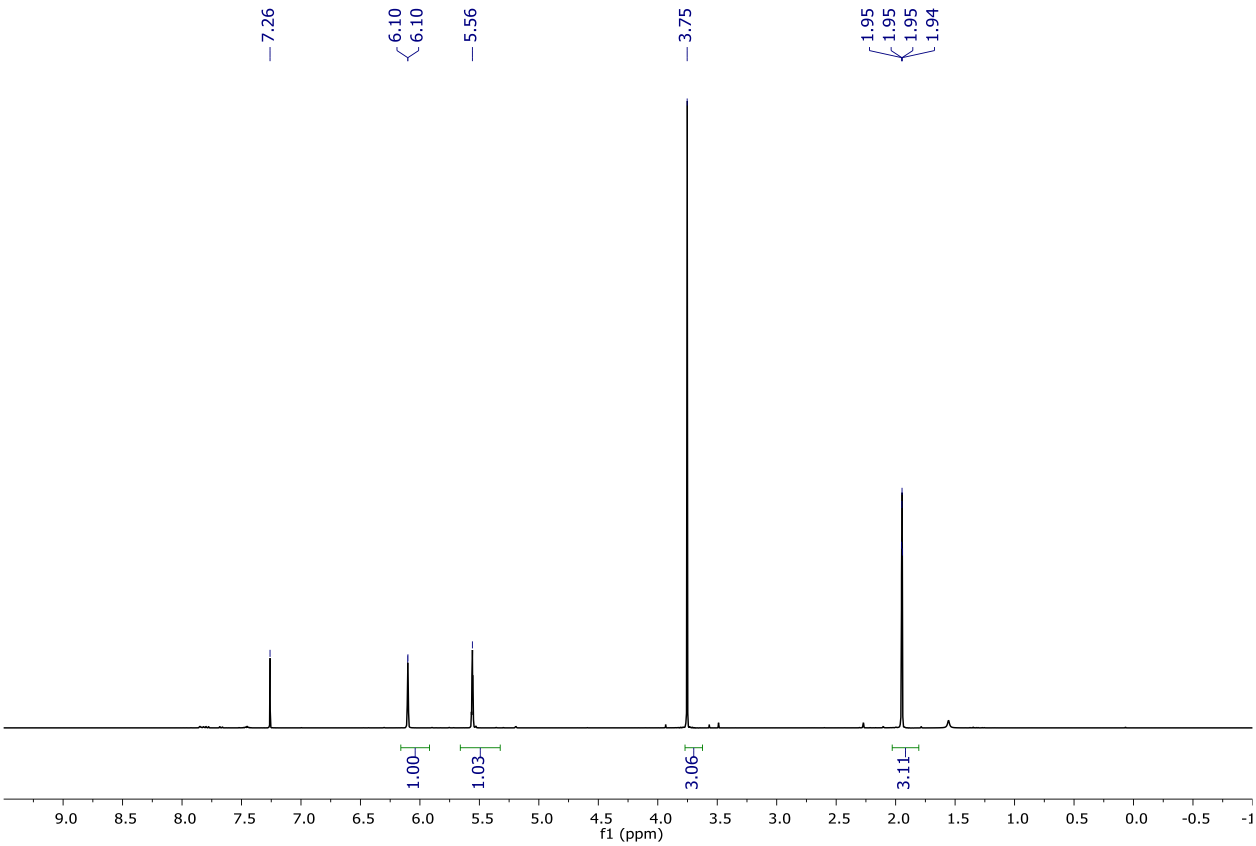

Figure S13. ^{1}H NMR spectrum of distillate after bulk depolymerization of **6**.

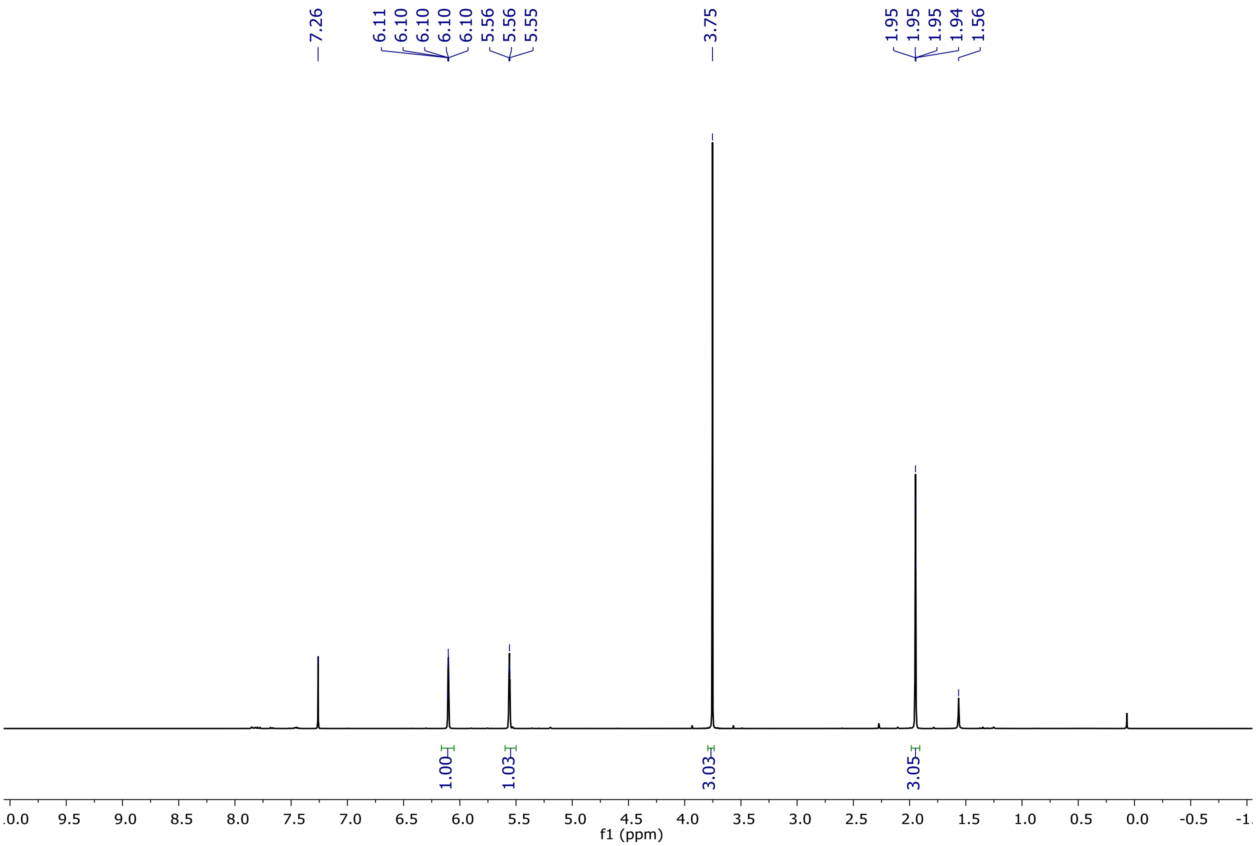

Figure S14. ^{1}H NMR spectrum of distillate after bulk depolymerization of **7**.

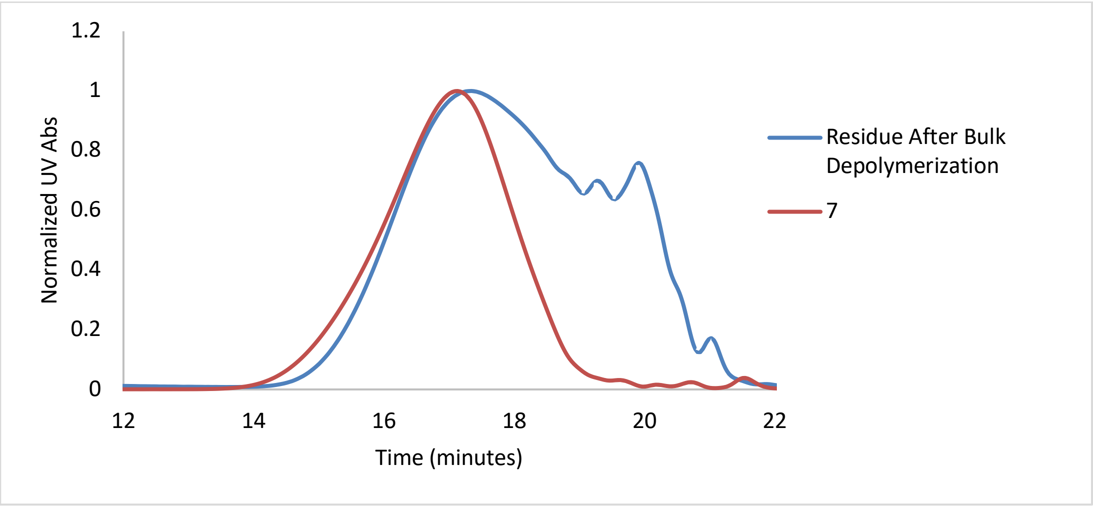

Figure S15. GPC spectrum before and after bulk depolymerization of **7**.

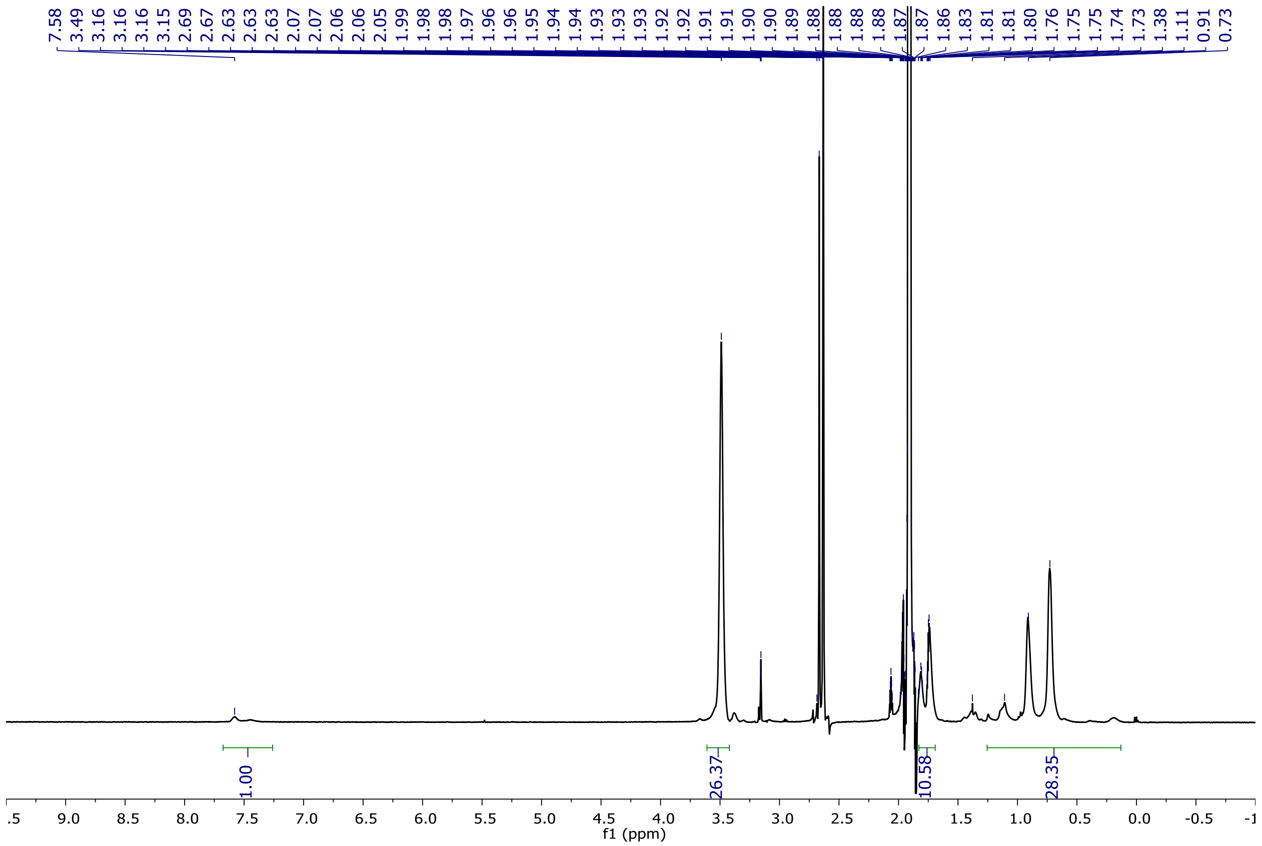

Figure S16. ^{1}H NMR spectrum of the repolymerization of the distillate after bulk depolymerization of **4** (Mw = 1.28 x 10^5 Da, Mn = 5.65 x 10^4, Đ = 2.3).

8. Stability Tests of P(MMA-co-AMS) Copolymers

For solution-phase stability tests, 1 mg of polymer was dissolved in 0.7 mL C_6D_6 and transferred to an oven-dried J-young tube. The J-young tube was placed into a heating block for a given amount of time at a given temperature. ^{1}H NMR were taken directly after cooling to room temperature.

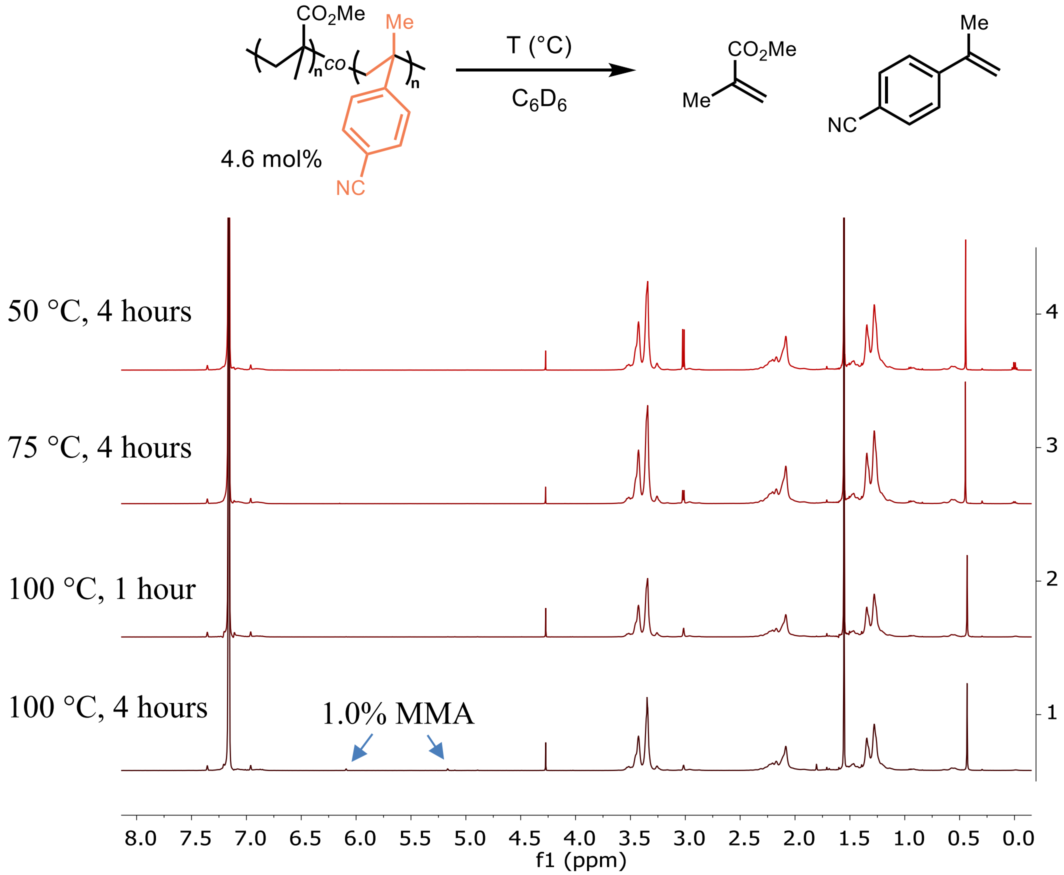

Figure S17. ^{1}H NMR spectra of the solution-phase stability tests of **4**.

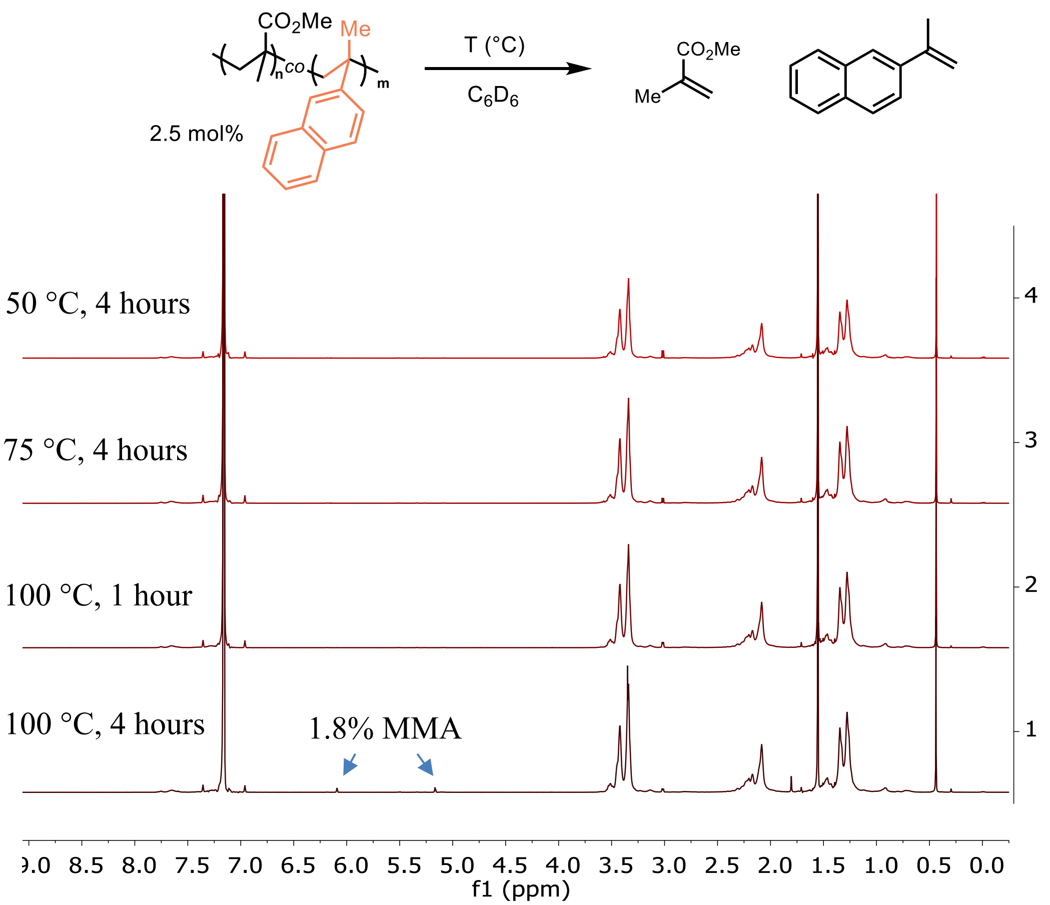

Figure S18. ^{1}H NMR spectra of the solution-phase stability tests of **6**.

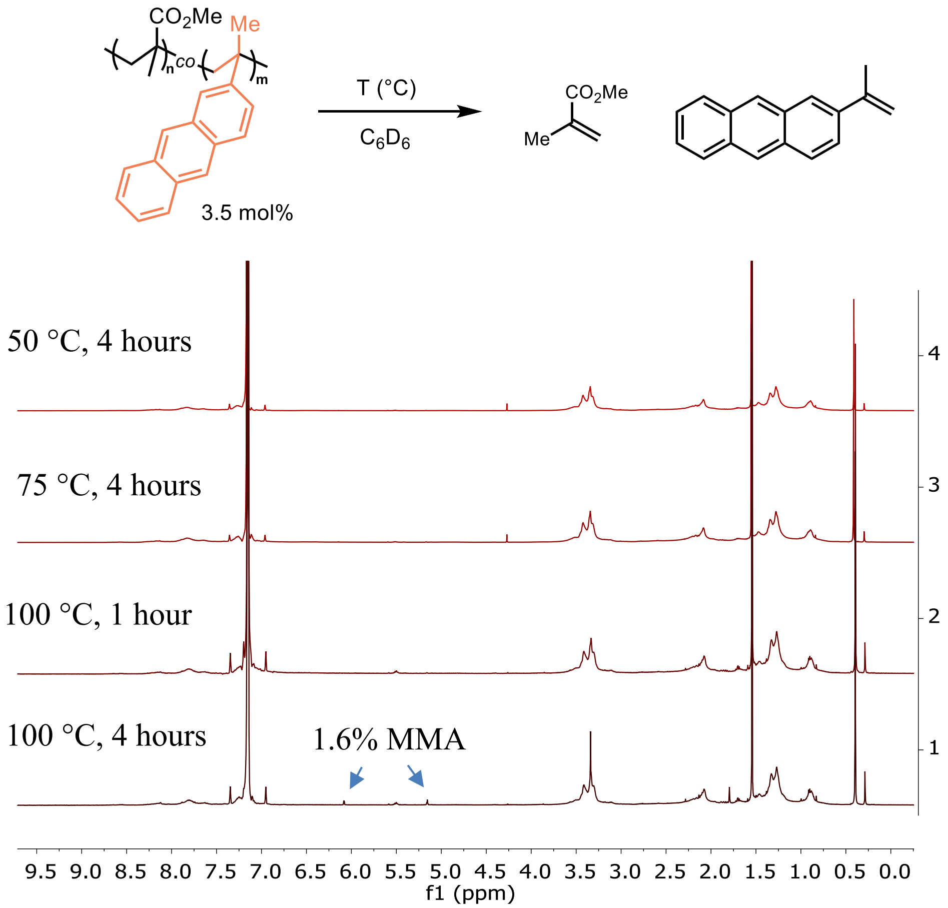

Figure S19. ^{1}H NMR spectra of the solution-phase stability tests of **7**.

For bulk stability tests, 10 mg of polymer was heated in a 4 mL vial for a given amount of time at a given temperature. GPC samples were prepared directly after allowing the reaction to cool to room temperature.

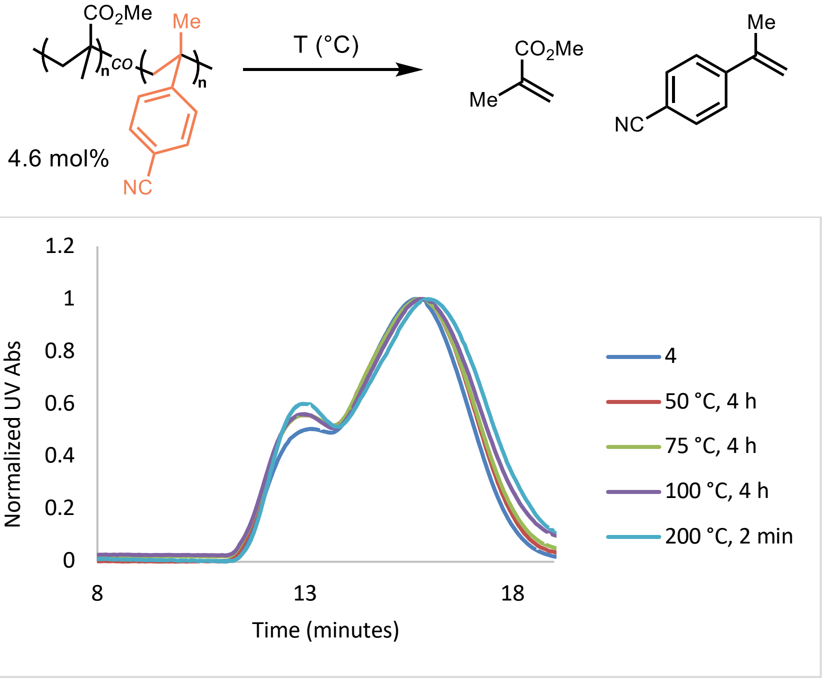

Figure S20. GPC spectra for the bulk stability tests of **4**.

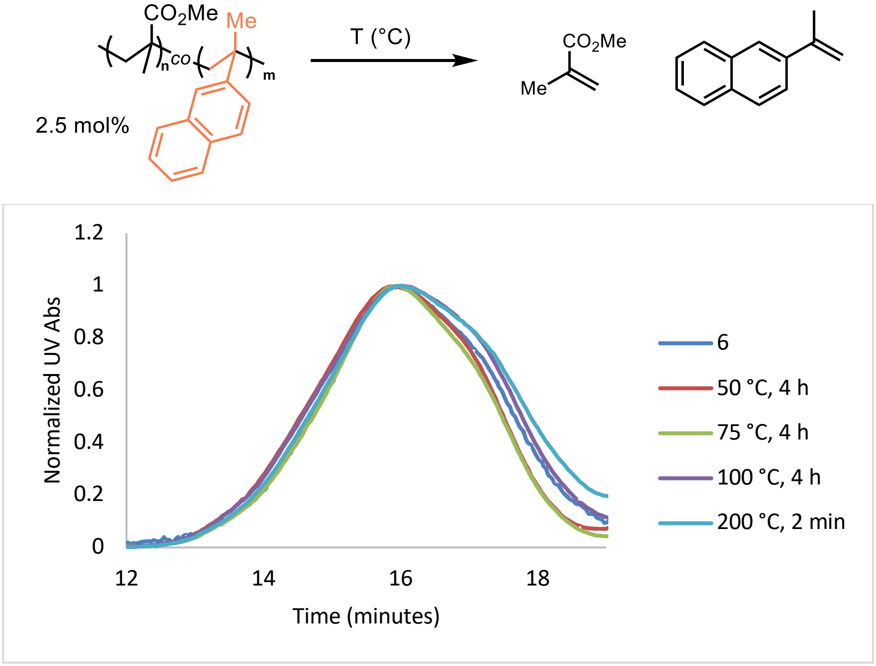

Figure S21. GPC spectra for the bulk stability tests of **6**.

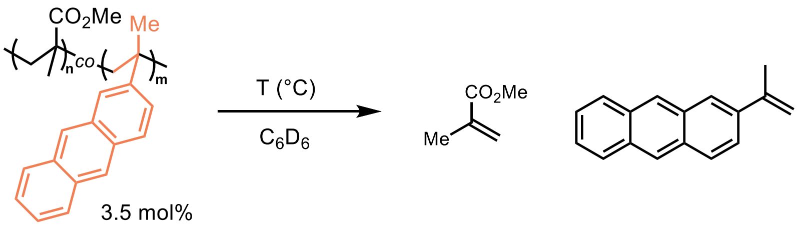

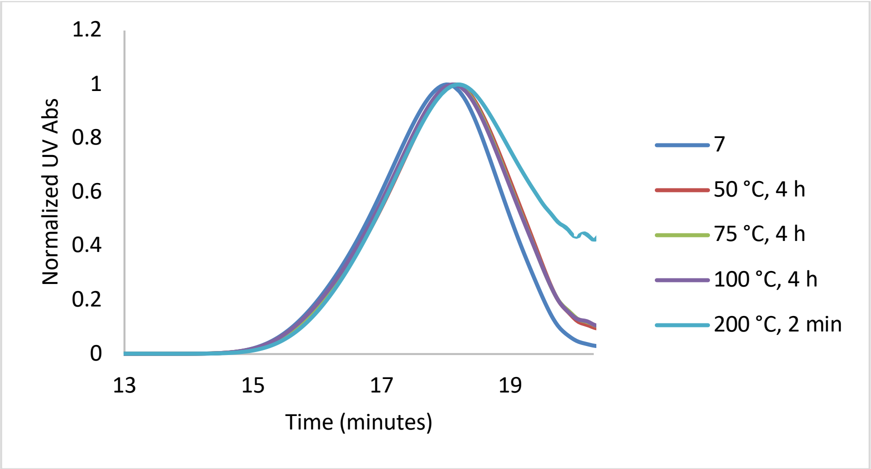

Figure S22. GPC spectra for the bulk stability tests of **7**.

9. TGA Thermograms

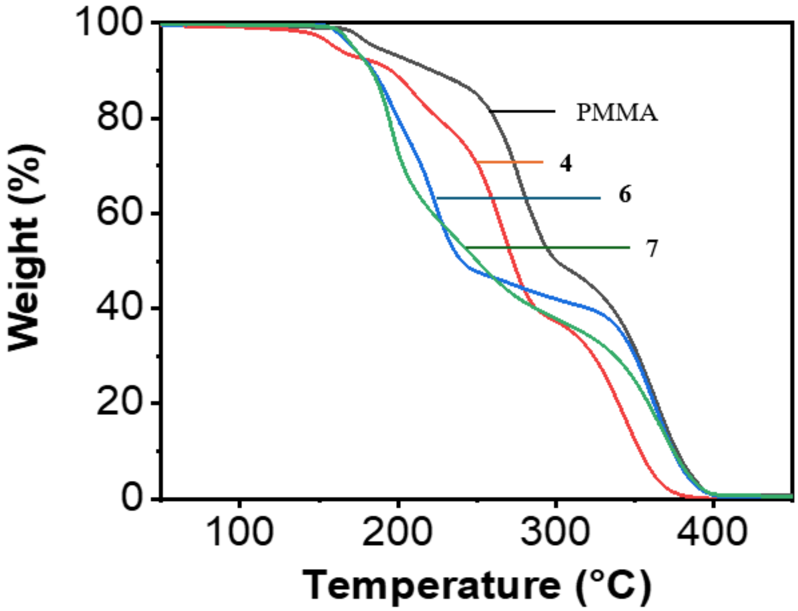

Figure S23. Comparison of TGA spectrum of **4**, **6**, **7**, and PMMA with 2 °C/minute heating ramp.

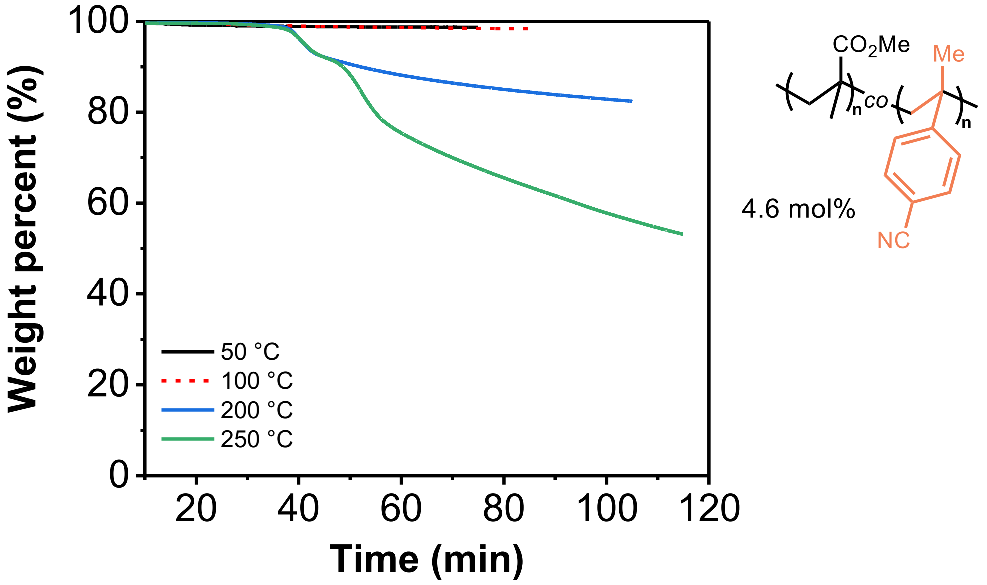

Figure S24. Isothermal TGA spectrum of **4**.

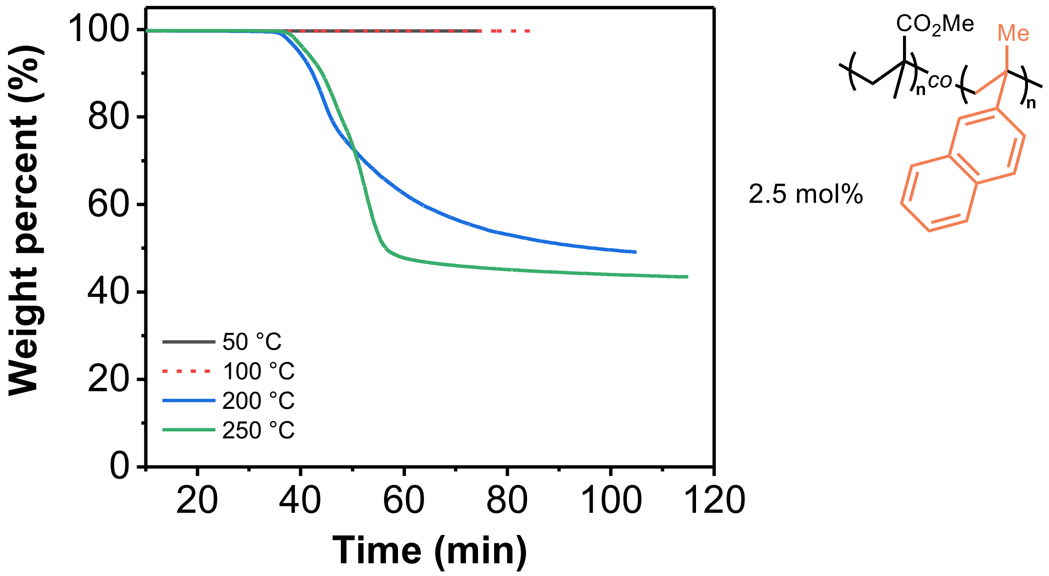

Figure S25. Isothermal TGA spectrum of **6**.

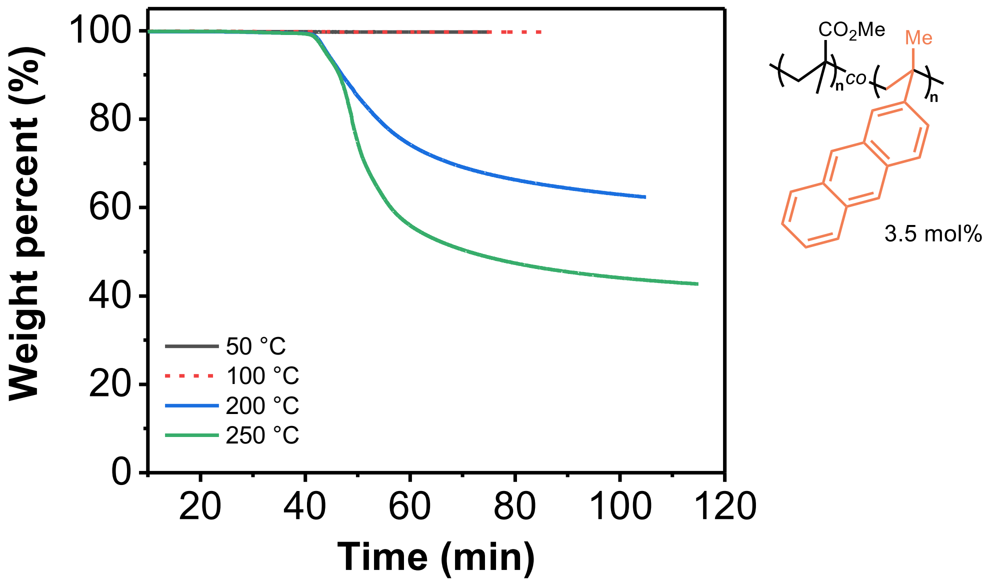

Figure S26. Isothermal TGA spectrum of **7**.

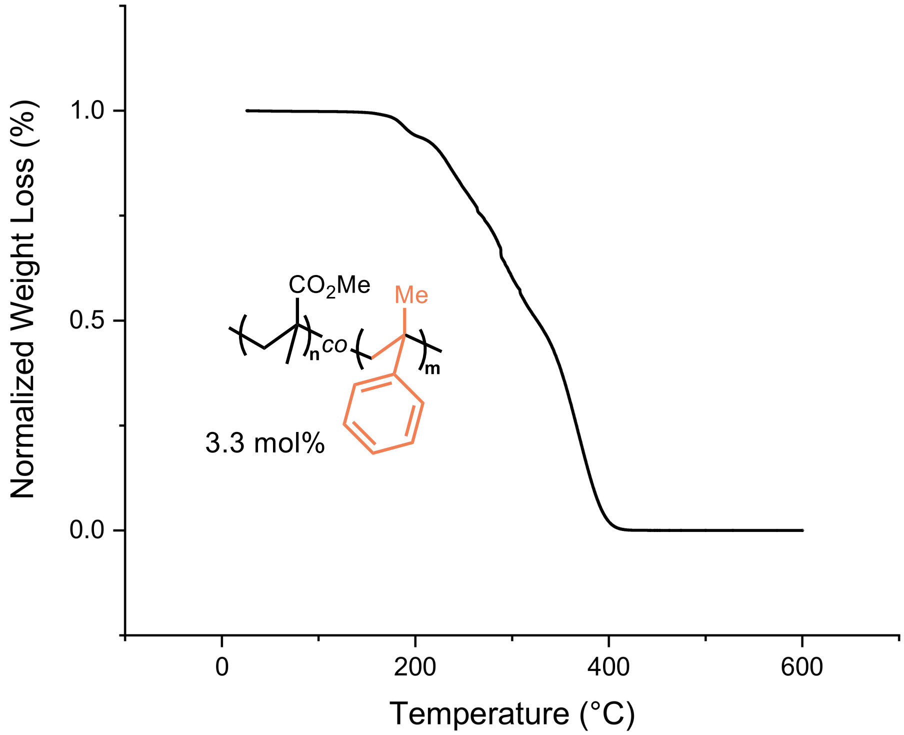

Figure S27. TGA thermogram of **2**.

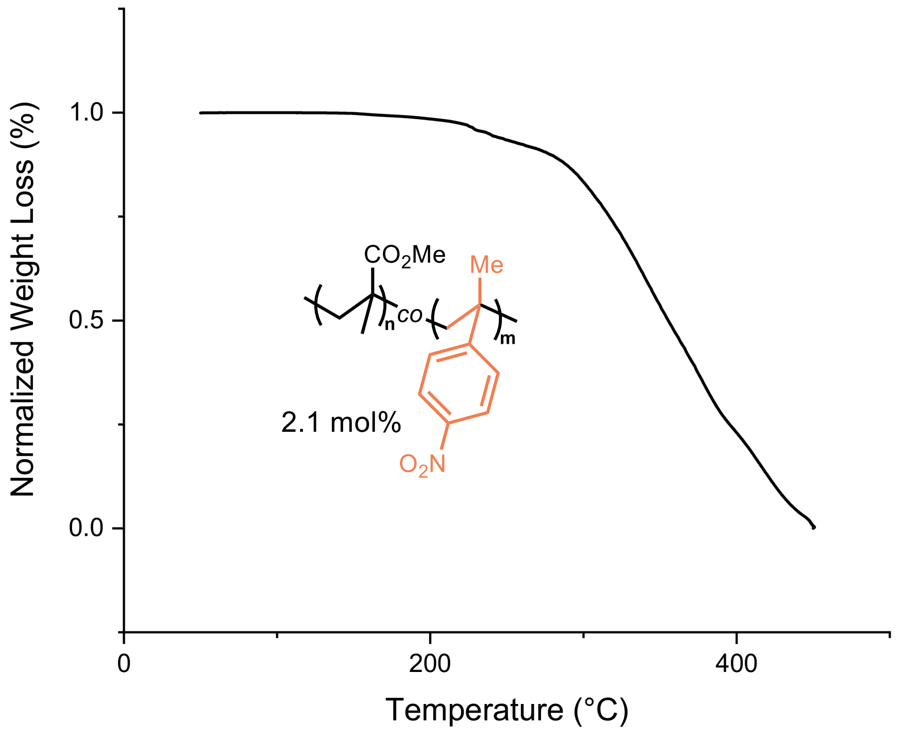

Figure S28. TGA thermogram of **3**.

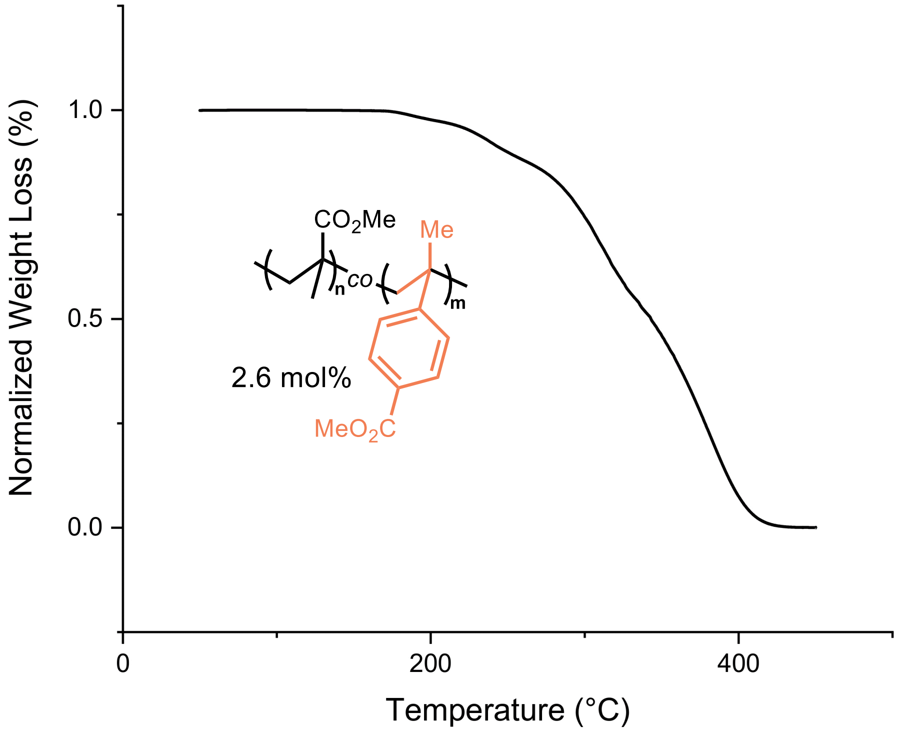

Figure S29. TGA thermogram of **5**.

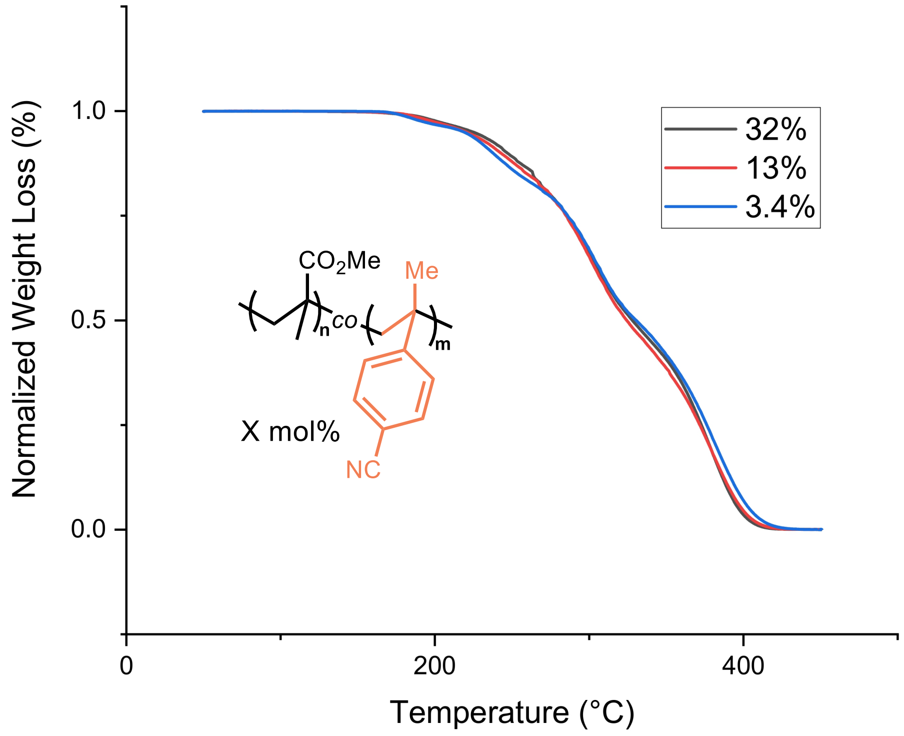

Figure S30. TGA thermograms of **4**.

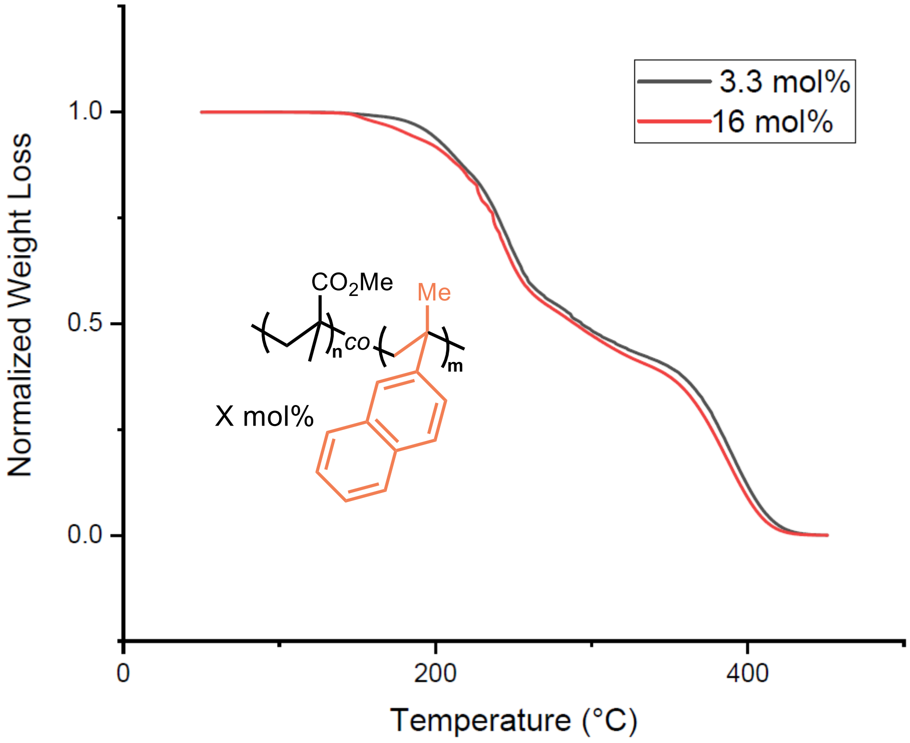

Figure S31. TGA thermograms of **6**.

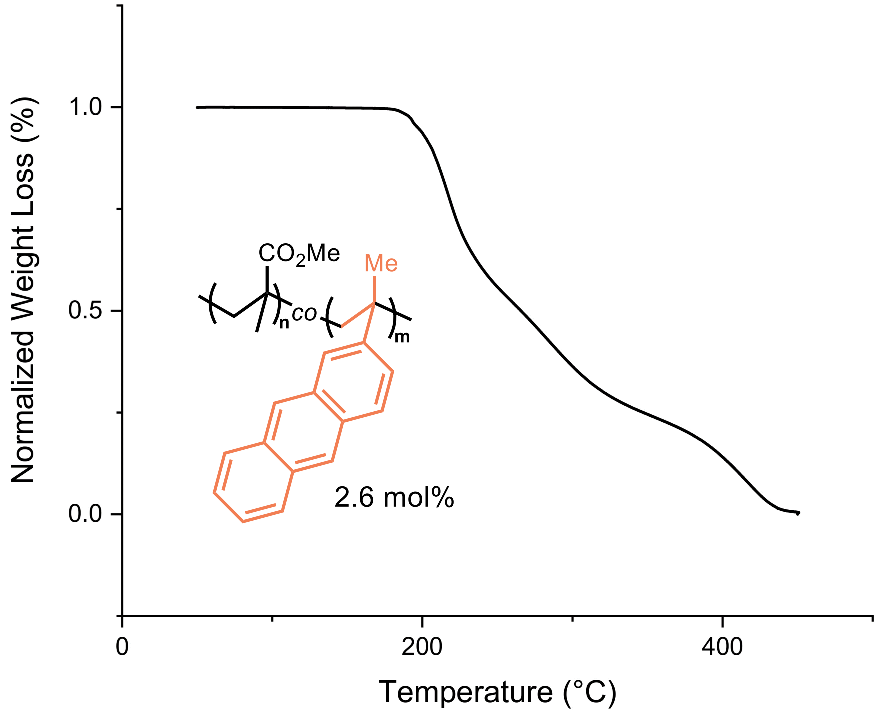

Figure S32. TGA thermogram of **7**.

10. Stress-Strain Curve

(a)

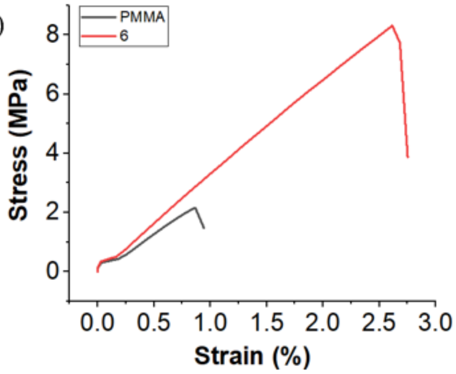

(b)

Sample	Young's Modulus/ Gpa	Elongation at break/%
PMMA	0.626	0.87
**6**	0.798	2.61

Figure S33. (a) Tensile stress-strain curve of **6** and pure PMMA films. (b) Young's modulus and Elongation at break of **6** and pure PMMA films.

Mechanical property of **6** was analyzed based on stress-strain curves. Both **6** and native PMMA polymer were prepared as thin films and cut into ~30 × 10 mm rectangular shape for testing, with a thickness of ~ 0.6 mm. The Young's modulus is 0.626 GPa for PMMA and 0.798 GPa for **6**. Their elongations at break are 0.87 and 2.61, respectively, which are also similar. Therefore, copolymerization would have minimal impact on the mechanical properties of PMMA.

11. NMR Spectra

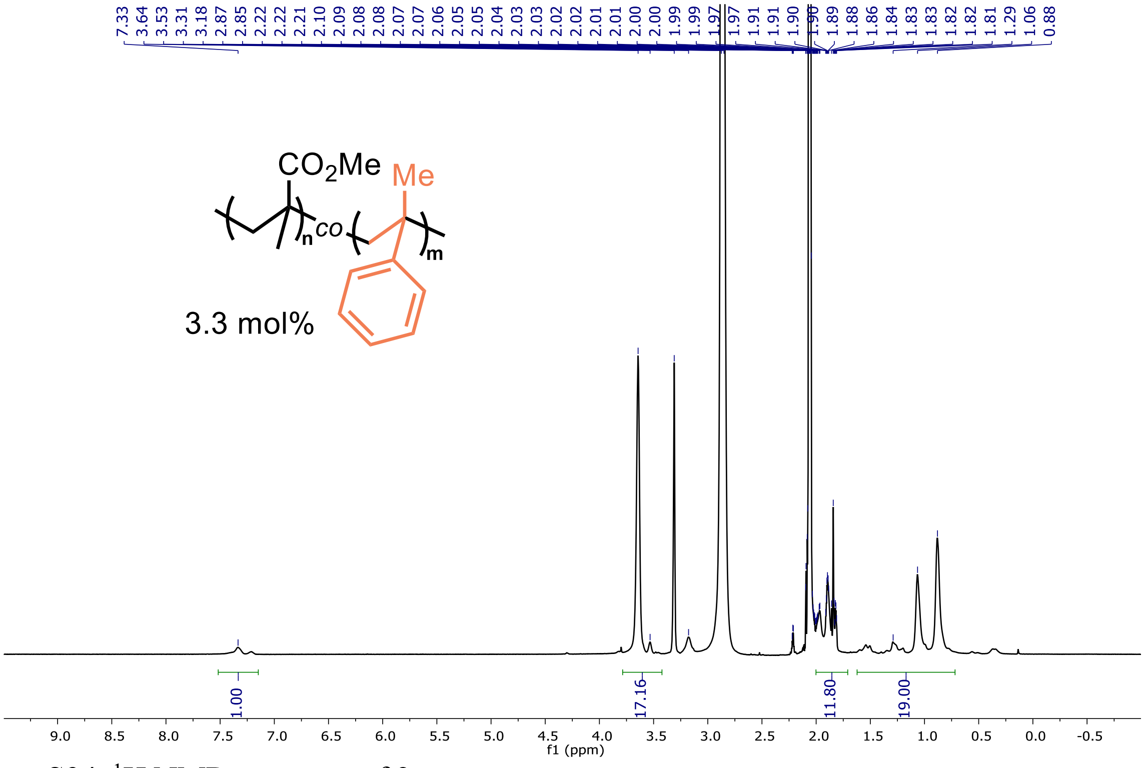

Figure S34. ^{1}H NMR spectrum of **2**.

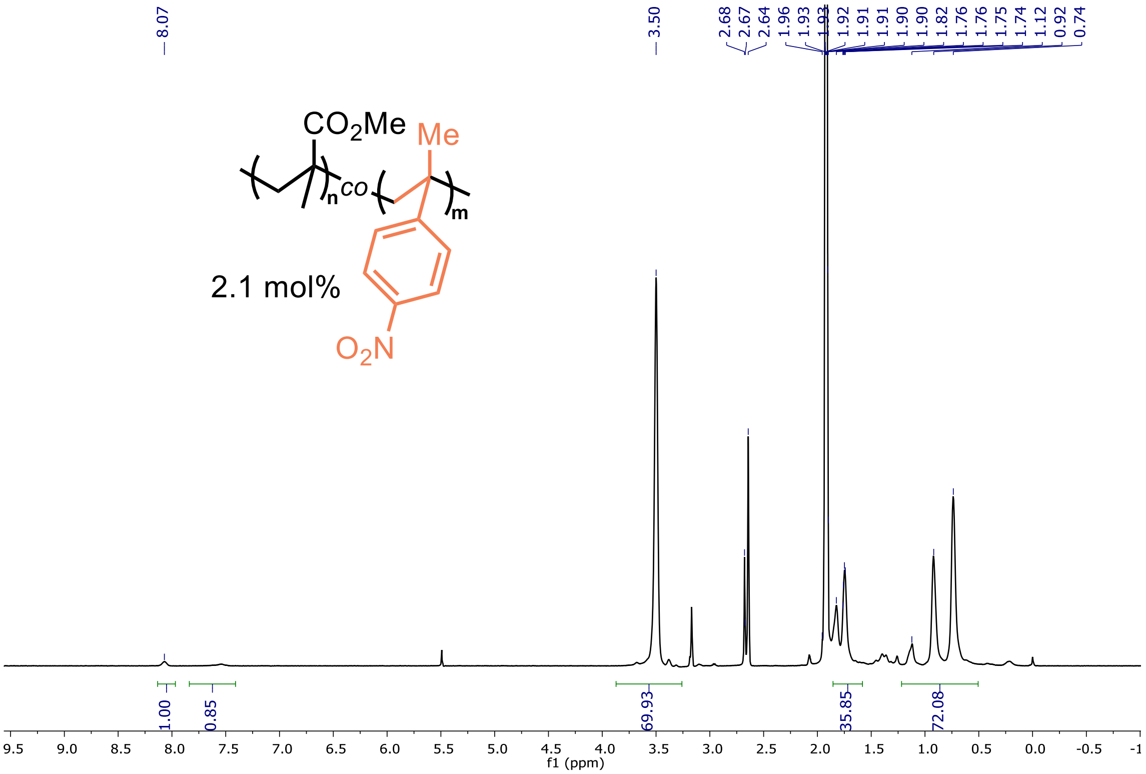

Figure S35. ^{1}H NMR spectrum of **3**.

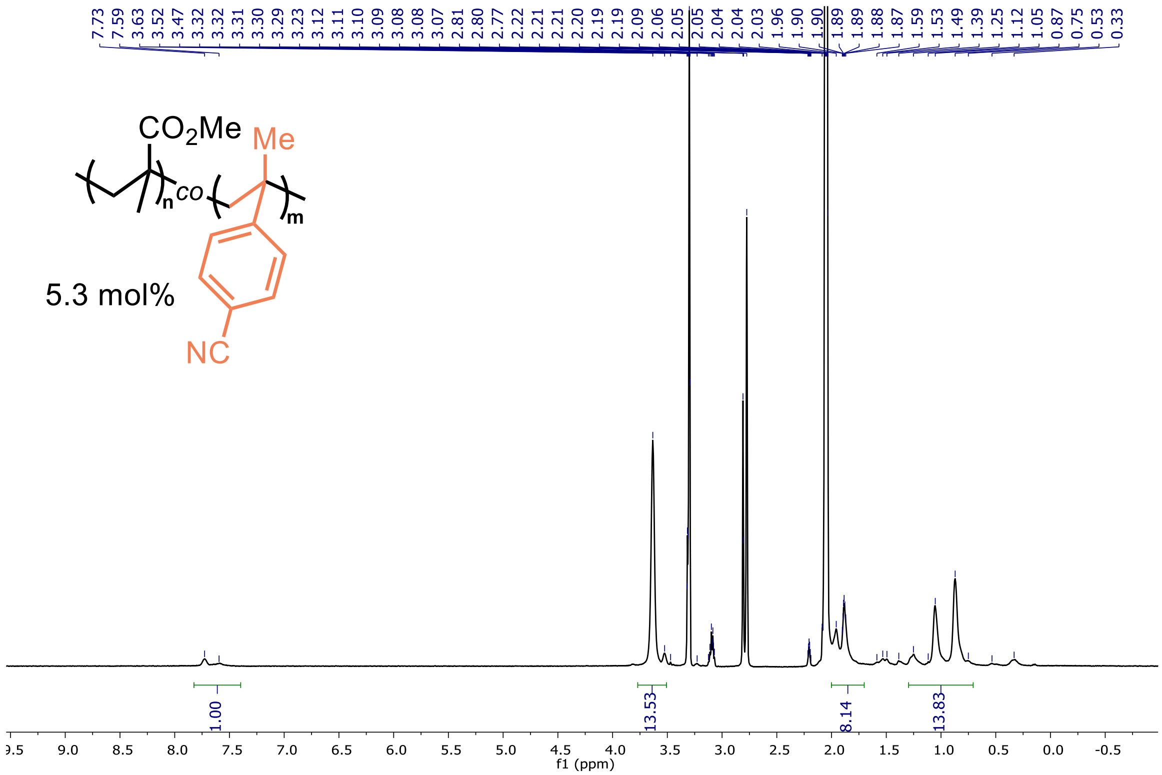

Figure S36. ^{1}H NMR spectrum of **4**.

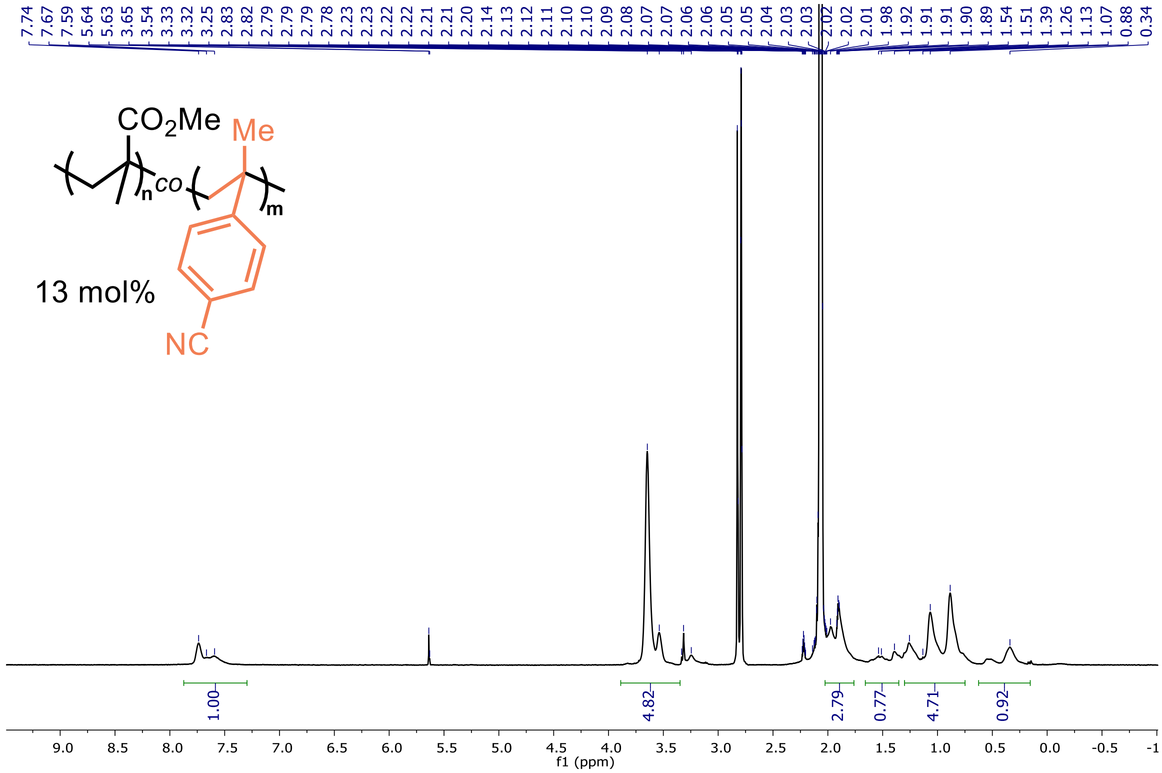

Figure S37. ^{1}H NMR spectrum of **4**.

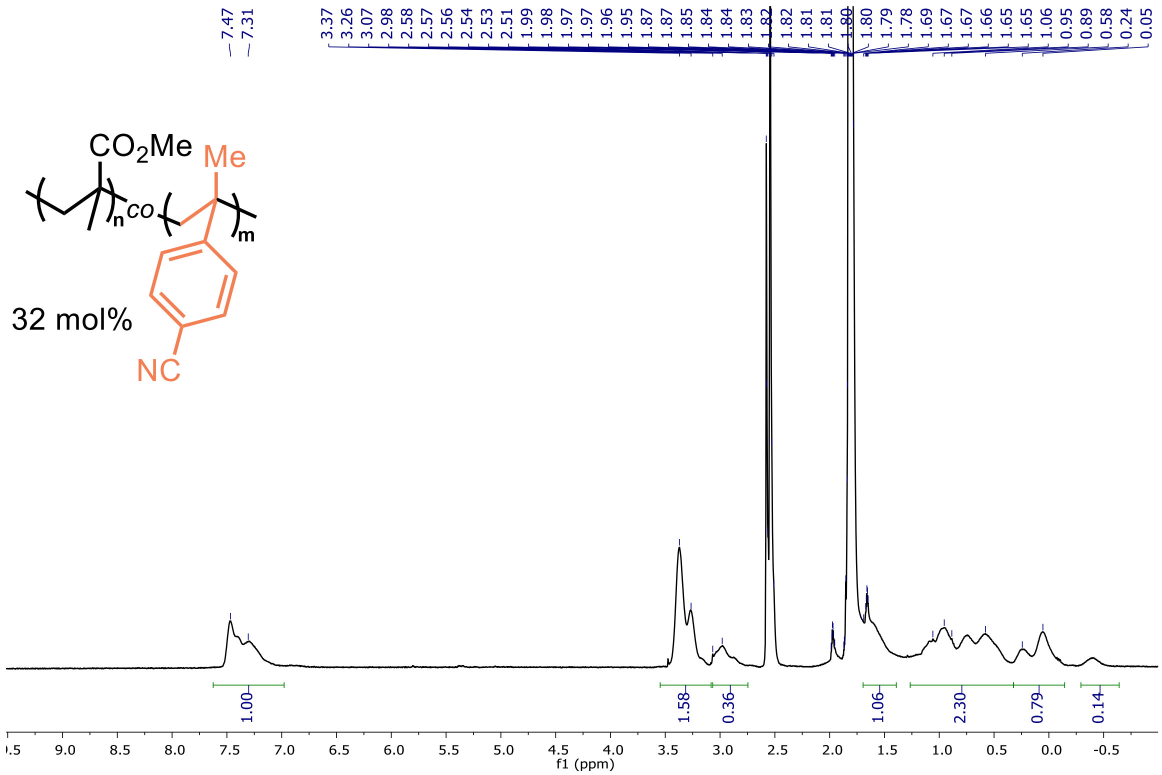

Figure S38. ^{1}H NMR spectrum of **4**.

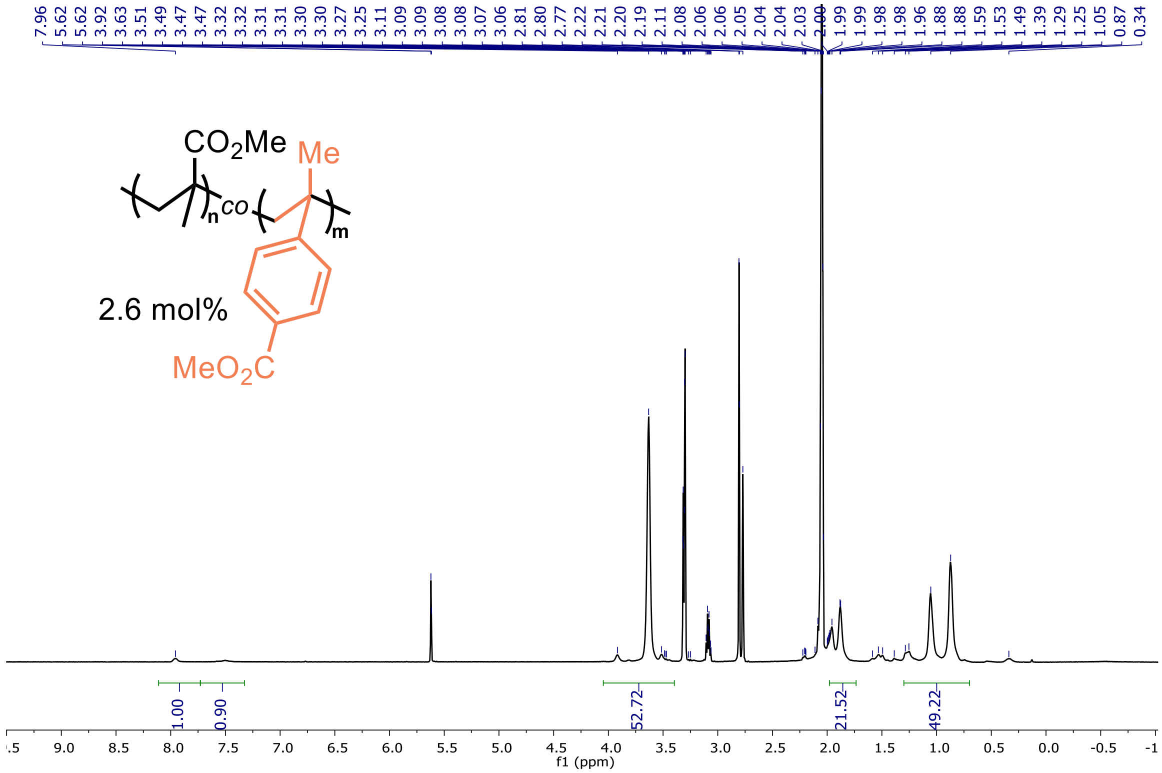

Figure S39. ^{1}H NMR spectrum of **5**.

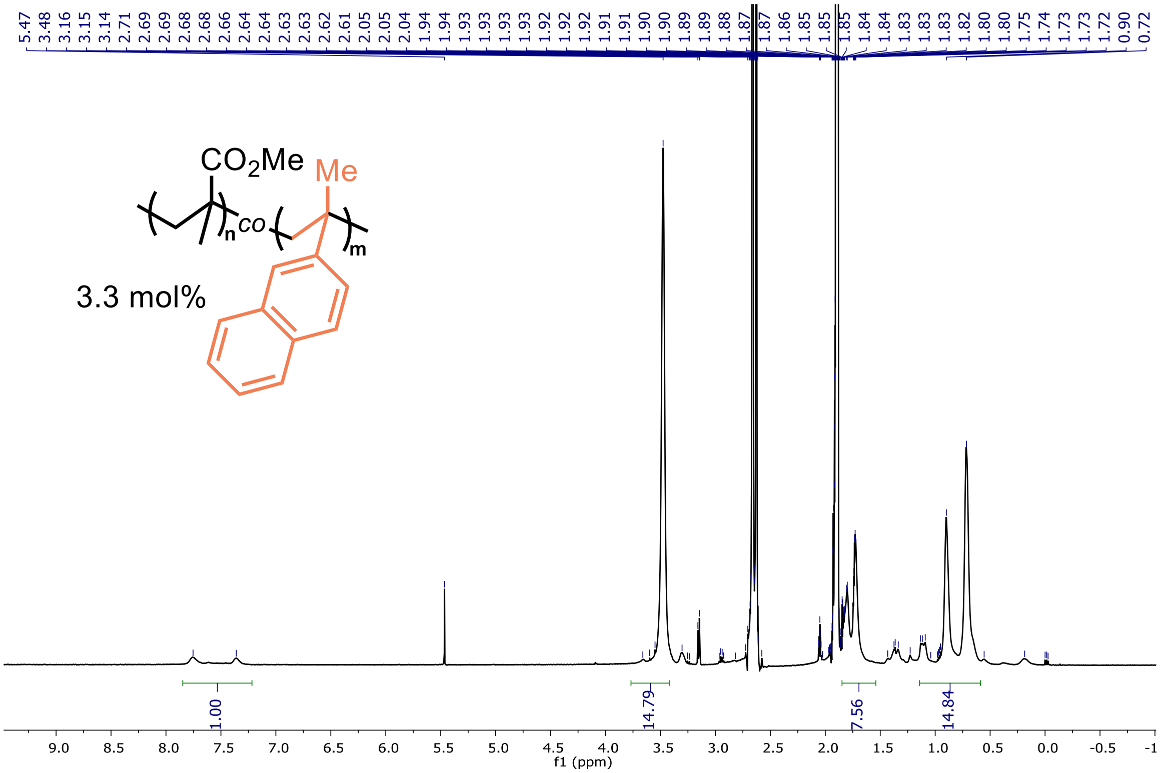

Figure S40. ^{1}H NMR spectrum of **6**.

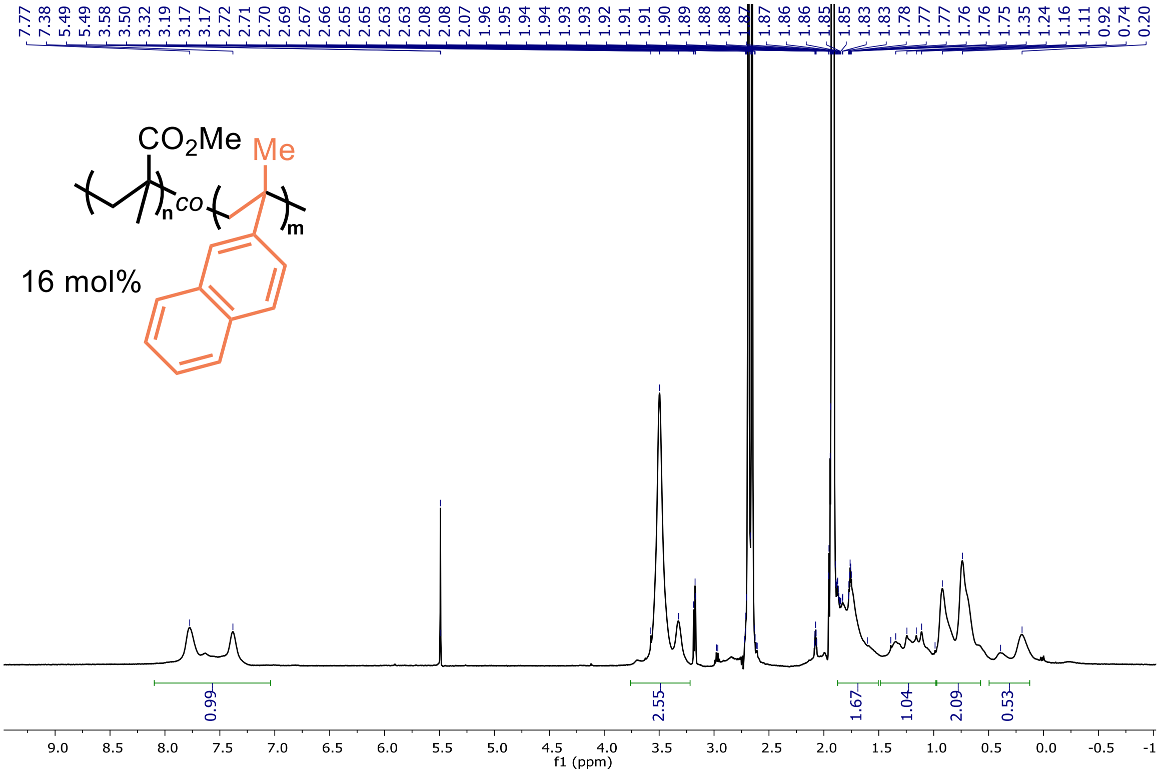

Figure S41. ^{1}H NMR spectrum of **6**.

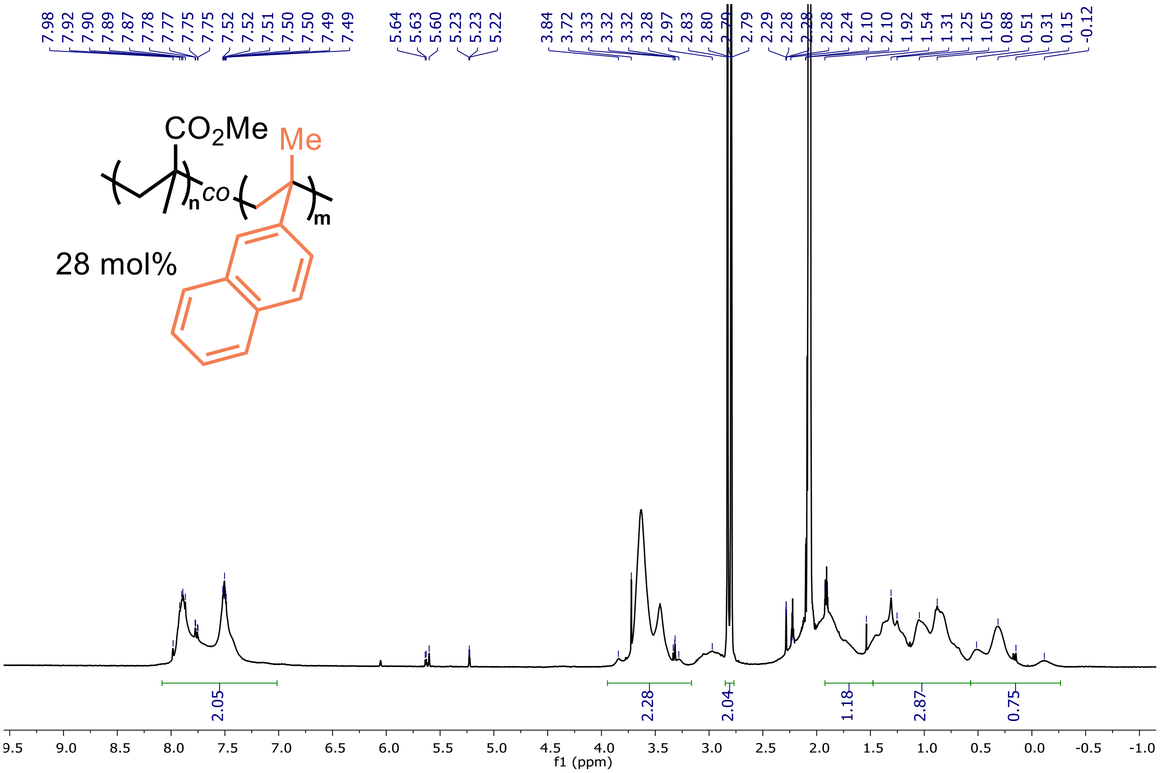

Figure S42. ^{1}H NMR spectrum of **6**.

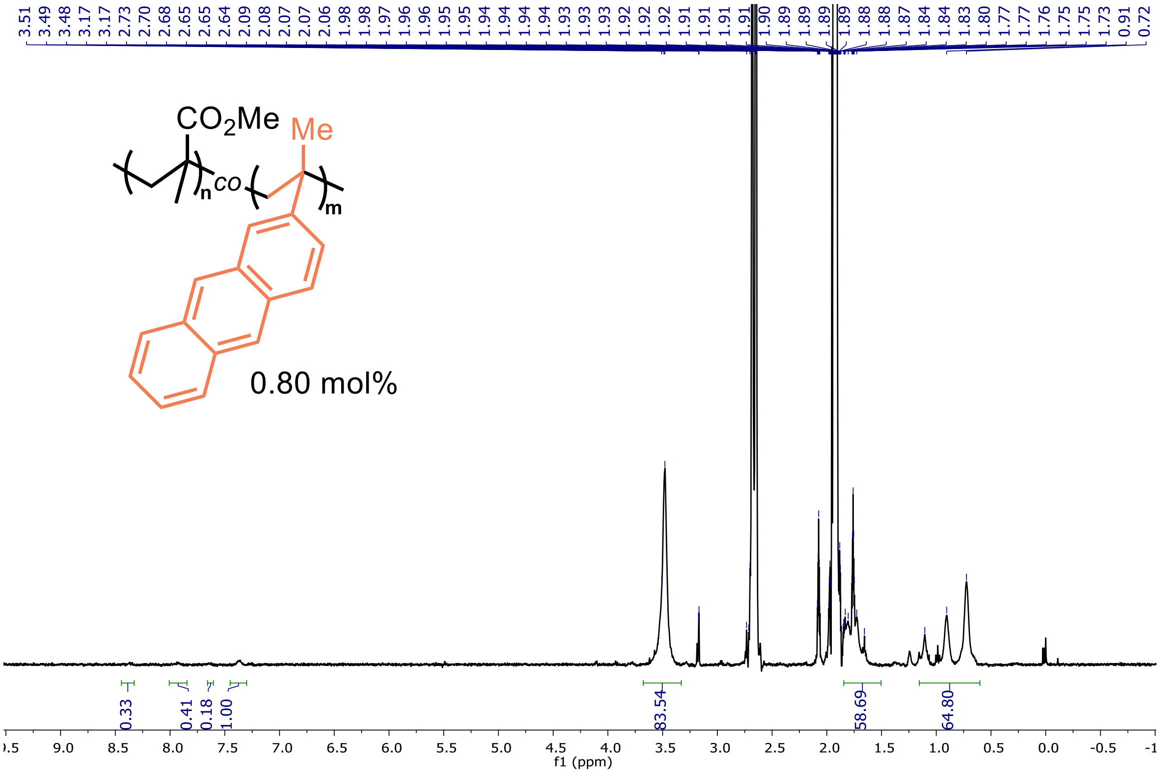

Figure S43. ^{1}H NMR spectrum of **7**.

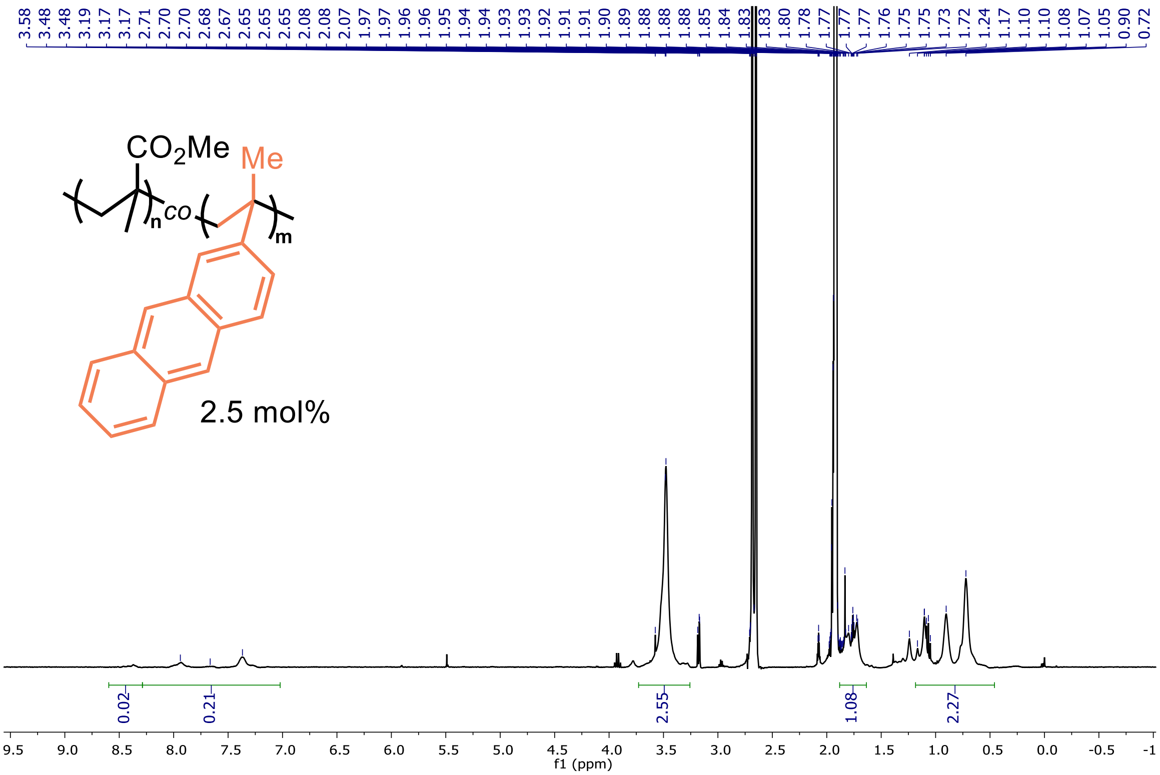

Figure S44. ^{1}H NMR spectrum of **7**.

12. DSC Spectra

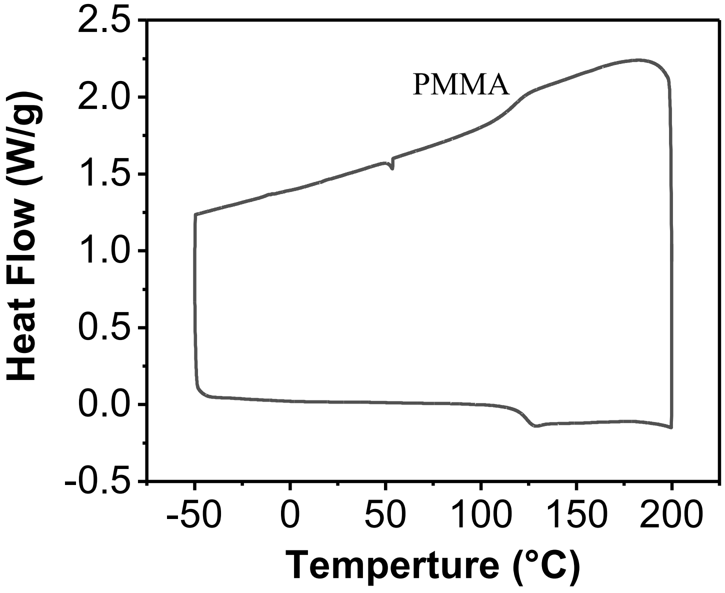

Figure S45. DSC spectrum of PMMA homopolymer.

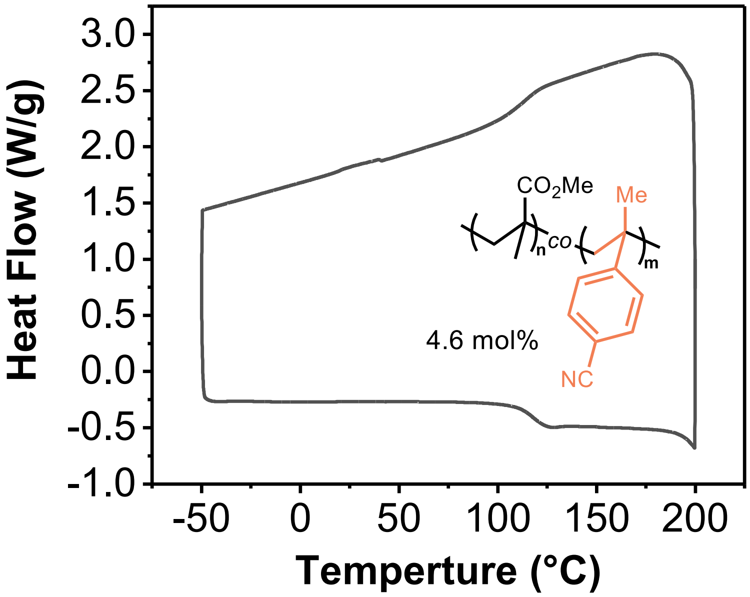

Figure S46. DSC spectrum of **4**.

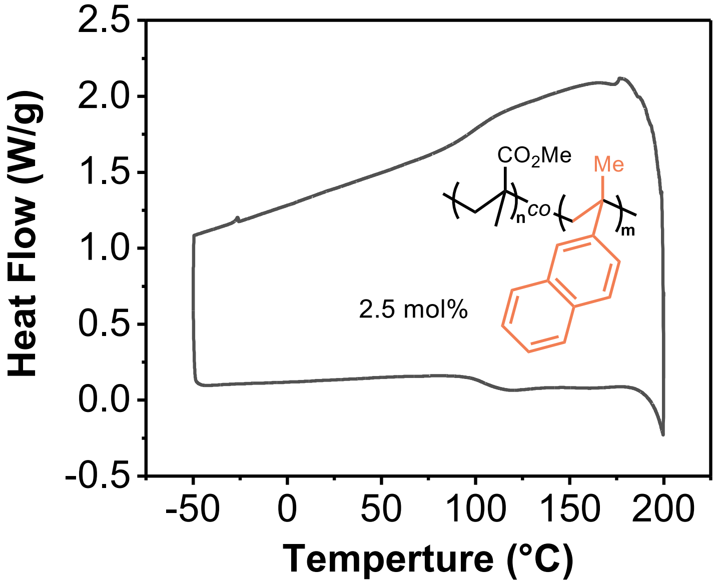

Figure S47. DSC spectrum of **6**.

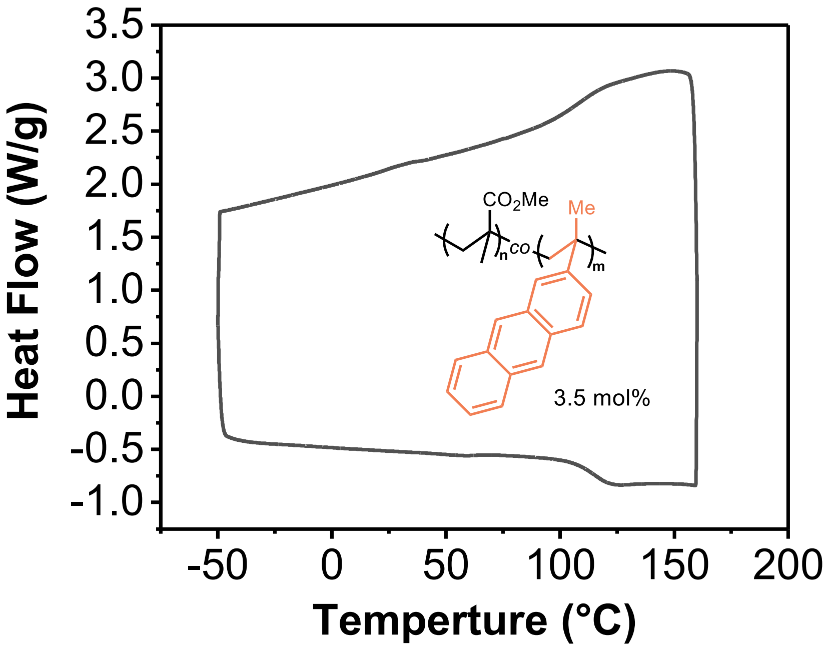

Figure S48. DSC spectrum of **7**.

13. DFT Calculations

Automated conformer sampling was performed for all molecules and radicals using CREST[6] and GFN2-xTB.[7] Cluster analysis using k-means clustering was used to generate 10 different populations of similar conformers. From each population the conformer lowest in free energy was selected for analysis by DFT. Density functional theory (DFT) calculations were performed using Gaussian 16.[8] All DFT geometry optimizations and vibrational frequency calculations were performed using the M06-2X functional[9] with the basis set def2-TZVP[10] for all atoms in the gas phase. The reported enthalpies include thermal corrections calculated at 298.15 K.

9

M06-2X/def2-TZVP SCF energy (au):	-732.115778245
M06-2X/def2-TZVP enthalpy (au):	-731.791496245
M06-2X/def2-TZVP free energy (au):	-731.854018245

Cartesian coordinates

ATOM	X	Y	Z
C	-2.053637	-0.257670	0.305471
O	-2.403789	-1.163667	1.018119
O	-2.649678	0.027492	-0.855770
C	-0.943639	0.724896	0.662008
C	-3.723111	-0.823472	-1.230440
H	-4.487847	-0.835190	-0.451192
H	-4.123675	-0.419493	-2.156555
H	-3.361705	-1.842762	-1.381751
C	-1.542260	1.597155	1.781149
H	-0.806845	2.322061	2.121981
H	-2.415362	2.135099	1.417637
H	-1.842575	0.977809	2.622944
C	-0.553474	1.619204	-0.510600
H	0.230186	2.305297	-0.196835
H	-0.195547	1.039303	-1.356193
H	-1.409946	2.201209	-0.842195
C	1.969368	0.006166	-0.610306
O	2.149302	-0.178136	-1.785696
O	2.567433	0.996155	0.068803
C	1.120581	-0.875997	0.296213
C	3.430075	1.832159	-0.687182
H	3.825105	2.570704	0.005898
H	4.240060	1.245319	-1.125041
H	2.876441	2.318417	-1.493333
C	2.126814	-1.674638	1.144463
H	1.595244	-2.386057	1.771794
H	2.811394	-2.225123	0.502420
H	2.704725	-1.008255	1.780248
C	0.310540	-1.846750	-0.560661
H	-0.303671	-2.480582	0.073234
H	-0.330554	-1.313082	-1.257009
H	0.981926	-2.471400	-1.145307
C	0.246848	-0.049972	1.257144
H	0.894290	0.662476	1.770833
H	-0.161373	-0.737452	2.00173

10

M06-2X/def2-TZVP SCF energy (au):	-888.900233791
M06-2X/def2-TZVP enthalpy (au):	-888.487943791
M06-2X/def2-TZVP free energy (au):	-888.556886791

Cartesian coordinates

ATOM X Y Z

C	-2.417993	2.079745	0.314299
O	-1.784313	3.080101	0.527985
O	-3.563238	1.796964	0.954147
C	-2.083589	1.035822	-0.742642
C	-3.983051	2.747707	1.920254
H	-4.928790	2.385623	2.315439
H	-3.240911	2.831256	2.717330
H	-4.110280	3.728555	1.457819
C	-2.193541	-0.399723	-0.192836
H	-2.185529	-1.069726	-1.053991
H	-3.174881	-0.490572	0.278236
C	-0.717506	1.355884	-1.345668
H	-0.750403	2.328162	-1.831674
H	0.050590	1.393251	-0.580149
H	-0.441844	0.603157	-2.079453
C	-3.160207	1.200417	-1.831450
H	-3.178926	2.226017	-2.194548
H	-2.940049	0.544340	-2.670609
H	-4.143671	0.952581	-1.439404
C	-1.144125	-0.896171	0.826964
C	-1.601571	-2.284836	1.314461
C	-1.115750	0.026216	2.047252
H	-1.731395	-2.974498	0.484822
H	-0.860720	-2.704698	1.991732
H	-2.548228	-2.206675	1.844904
H	-0.743548	1.015601	1.796169
H	-2.123216	0.128507	2.445344
H	-0.488974	-0.395266	2.830606
H	1.337389	0.100074	1.591367
C	1.369208	-0.526301	0.713740
C	0.226123	-1.078504	0.194383
C	0.350489	-1.896106	-0.951291
C	1.557863	-2.121778	-1.545115
C	2.737660	-1.547856	-1.030587
C	2.634143	-0.735700	0.125958
C	3.805988	-0.157185	0.656817
C	5.020134	-0.373971	0.067979
C	5.120291	-1.179141	-1.077451
C	4.003096	-1.754105	-1.615538
H	-0.528789	-2.360218	-1.373084
H	1.625207	-2.750163	-2.422490
H	4.073083	-2.375414	-2.497492
H	6.088576	-1.339302	-1.529176
H	5.913295	0.073566	0.479474
H	3.726107	0.463479	1.538312

11

M06-2X/def2-TZVP SCF energy (au): -1042.52880944

M06-2X/def2-TZVP enthalpy (au): -1042.06689344

M06-2X/def2-TZVP free energy (au): -1042.14206144

Cartesian coordinates

ATOM	X	Y	Z
C	-3.583896	1.868271	-0.187447
O	-3.007765	2.922272	-0.259839
O	-4.718956	1.709321	0.510337
C	-3.180410	0.594097	-0.916956
C	-5.198060	2.863681	1.182278
H	-6.140841	2.580799	1.643741
H	-4.481066	3.185114	1.940981
H	-5.346520	3.681307	0.474076
C	-3.202000	-0.635994	0.011783
H	-3.131765	-1.516363	-0.629220
H	-4.183782	-0.661896	0.490071
C	-1.834571	0.818785	-1.603166
H	-1.500808	-0.093166	-2.091013
H	-1.930480	1.603794	-2.349611
H	-1.078093	1.129829	-0.890173
C	-4.259428	0.380069	-1.994633
H	-4.339936	1.259323	-2.630590
H	-3.994300	-0.470415	-2.618745
H	-5.227754	0.192615	-1.536966
C	-2.142075	-0.755443	1.130138
C	-2.500070	-1.998537	1.967833
C	-2.217392	0.455934	2.061547
H	-3.463919	-1.862151	2.453684
H	-2.552506	-2.892501	1.352323
H	-1.746650	-2.159455	2.736124
H	-3.239088	0.575920	2.416088
H	-1.577324	0.310771	2.929534
H	-1.918918	1.370432	1.556614
C	5.467496	-1.186968	-1.235499
C	4.171040	-0.964790	-0.705837
C	3.085185	-1.769907	-1.040399
C	3.977798	0.124917	0.200301
C	2.709697	0.355822	0.726683
C	1.623781	-0.447058	0.389131
C	1.819076	-1.534535	-0.515174
C	6.510504	-0.382498	-0.892888
C	5.091158	0.938501	0.531745
H	2.565192	1.181443	1.410349
C	0.327750	-0.209794	0.913220
C	0.695356	-2.335184	-0.837621
C	-0.530534	-2.068513	-0.315624
C	-0.747253	-0.979794	0.571072
H	0.226973	0.624289	1.589574
H	0.834097	-3.168541	-1.512363
H	-1.359990	-2.705761	-0.584241
C	6.320047	0.692651	0.001137
H	5.607599	-2.011713	-1.920178

H	3.229271	-2.594696	-1.725258
H	7.495076	-0.557782	-1.301701
H	4.939465	1.761008	1.216525
H	7.162881	1.317843	0.258054

Radical **9a**

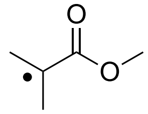

M06-2X/def2-TZVP SCF energy (au):	-346.348372229
M06-2X/def2-TZVP enthalpy (au):	-346.204587229
M06-2X/def2-TZVP free energy (au):	-346.249088229

Cartesian coordinates

ATOM	X	Y	Z
C	-0.313457	0.000000	0.013348
O	-0.798950	0.879518	-0.644558
O	0.923820	-0.471434	-0.218446
C	-0.967161	-0.662053	1.219237
C	1.600912	0.119243	-1.323885
H	2.572029	-0.364211	-1.373403
H	1.039987	-0.050092	-2.242913
H	1.712181	1.192037	-1.173681
C	-2.402842	-0.159413	1.337620
H	-2.907618	-0.671422	2.158817
H	-2.408619	0.912980	1.531843
H	-2.971513	-0.332974	0.426703
C	-0.165545	-0.185875	2.445360
H	-0.127127	0.904227	2.482336
H	-0.655125	-0.539313	3.354184
H	0.853719	-0.571217	2.423848

Radical **9b**

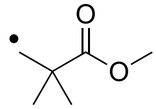

M06-2X/def2-TZVP SCF energy (au):	-385.635991679
M06-2X/def2-TZVP enthalpy (au):	-385.463873679
M06-2X/def2-TZVP free energy (au):	-385.510615679

Cartesian coordinates

ATOM	X	Y	Z
C	-0.554577	0.000000	-0.363198
O	-1.087839	0.495945	-1.318078
O	0.680877	-0.526403	-0.423086
C	-1.138645	-0.056125	1.042413
C	1.294748	-0.488673	-1.707983
H	2.272696	-0.944862	-1.586337
H	0.696620	-1.047820	-2.427385
H	1.390026	0.539185	-2.054826

C	-0.995315	-1.456614	1.674037
H	-1.184965	-1.336007	2.743802
H	0.053315	-1.750090	1.584142
C	-2.578059	0.447694	0.996751
H	-2.598520	1.492933	0.688792
H	-3.184895	-0.115960	0.291321
H	-3.032614	0.359508	1.984998
C	-0.292911	0.915456	1.887003
H	-0.276433	1.908131	1.433649
H	-0.732961	1.003640	2.881546
H	0.732482	0.560064	1.987730

Radical **10a**

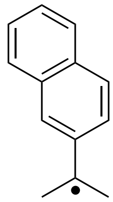

M06-2X/def2-TZVP SCF energy (au): -503.140322950
M06-2X/def2-TZVP enthalpy (au): -502.908808950
M06-2X/def2-TZVP free energy (au): -502.959142950

Cartesian coordinates

ATOM	X	Y	Z
C	-1.115938	-0.860222	0.825484
C	-1.611674	-2.225484	1.337428
C	-1.056019	0.088348	2.023177
H	-1.762476	-2.929923	0.518218
H	-0.885604	-2.658916	2.026876
H	-2.562155	-2.107808	1.861719
H	-0.649099	1.066934	1.763610
H	-2.061504	0.233466	2.422505
H	-0.439258	-0.333190	2.818747
H	1.378819	0.124112	1.554768
C	1.408091	-0.531722	0.693912
C	0.258640	-1.082521	0.194654
C	0.370632	-1.934820	-0.939188
C	1.572008	-2.186240	-1.532451
C	2.764647	-1.608645	-1.032874
C	2.675365	-0.769945	0.103488
C	3.859346	-0.186855	0.616008
C	5.068719	-0.428290	0.026688
C	5.155536	-1.266454	-1.106436
C	4.028910	-1.842545	-1.622932
H	-0.518722	-2.399997	-1.345641
H	1.629121	-2.837435	-2.396914
H	4.087555	-2.486684	-2.492369
H	6.119242	-1.449660	-1.563514
H	5.967154	0.023474	0.426840
H	3.789426	0.456683	1.485031

Radical **10b**

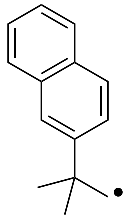

M06-2X/def2-TZVP SCF energy (au): -542.423570393
M06-2X/def2-TZVP enthalpy (au): -542.163486393
M06-2X/def2-TZVP free energy (au): -542.215606393

Cartesian coordinates

ATOM	X	Y	Z
C	-2.157943	-0.365404	-0.206352
H	-2.114247	-1.014658	-1.084687
H	-3.147268	-0.516292	0.232171
C	-1.115938	-0.860222	0.825484
C	-1.611674	-2.225484	1.337428
C	-1.056019	0.088348	2.023177
H	-1.762476	-2.929923	0.518218
H	-0.885604	-2.658916	2.026876
H	-2.562155	-2.107808	1.861719
H	-0.649099	1.066934	1.763610
H	-2.061504	0.233466	2.422505
H	-0.439258	-0.333190	2.818747
H	1.378819	0.124112	1.554768
C	1.408091	-0.531722	0.693912
C	0.258640	-1.082521	0.194654
C	0.370632	-1.934820	-0.939188
C	1.572008	-2.186240	-1.532451
C	2.764647	-1.608645	-1.032874
C	2.675365	-0.769945	0.103488
C	3.859346	-0.186855	0.616008
C	5.068719	-0.428290	0.026688
C	5.155536	-1.266454	-1.106436
C	4.028910	-1.842545	-1.622932
H	-0.518722	-2.399997	-1.345641
H	1.629121	-2.837435	-2.396914
H	4.087555	-2.486684	-2.492369
H	6.119242	-1.449660	-1.563514
H	5.967154	0.023474	0.426840
H	3.789426	0.456683	1.485031

Radical **11a**

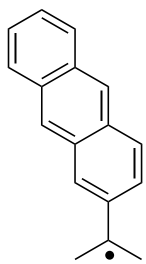

M06-2X/def2-TZVP SCF energy (au): -656.770481077
M06-2X/def2-TZVP enthalpy (au): -656.489426077
M06-2X/def2-TZVP free energy (au): -656.546151077

Cartesian coordinates

ATOM	X	Y	Z
C	-2.105938	-0.694101	1.108308
C	-2.484800	-1.913693	1.969527
C	-2.159364	0.541158	2.007387
H	-3.453785	-1.750373	2.445374
H	-2.552062	-2.823215	1.370932
H	-1.737764	-2.075071	2.748137
H	-3.181850	0.691550	2.358443
H	-1.522176	0.407893	2.883313
H	-1.839432	1.448646	1.492793
C	5.534026	-1.297617	-1.207347
C	4.228249	-1.036503	-0.694383
C	3.132101	-1.834023	-1.016483
C	4.043565	0.082703	0.178118
C	2.773850	0.348781	0.686690
C	1.679851	-0.448705	0.365234
C	1.866387	-1.564207	-0.508026
C	6.584367	-0.502362	-0.877576
C	5.173502	0.893451	0.499014
H	2.635740	1.198048	1.347189
C	0.373370	-0.180202	0.878632
C	0.726771	-2.364645	-0.820292
C	-0.494177	-2.070294	-0.310570
C	-0.704186	-0.945933	0.556302
H	0.269218	0.679826	1.527704
H	0.857562	-3.219252	-1.473679
H	-1.333001	-2.705828	-0.564983
C	6.400709	0.612012	-0.009921
H	5.668687	-2.146241	-1.867435
H	3.269447	-2.682597	-1.678074
H	7.570088	-0.710102	-1.273105
H	5.029352	1.740583	1.158939
H	7.249732	1.234642	0.240612

Radical **11b**

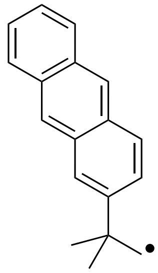

M06-2X/def2-TZVP SCF energy (au): -696.052259373
M06-2X/def2-TZVP enthalpy (au): -695.742508373
M06-2X/def2-TZVP free energy (au): -695.800865373

Cartesian coordinates

ATOM	X	Y	Z
C	-3.166566	-0.594945	-0.014859
H	-3.046985	-1.450926	-0.684250
H	-4.146872	-0.712103	0.453264
C	-2.105938	-0.694101	1.108308
C	-2.484800	-1.913693	1.969527
C	-2.159364	0.541158	2.007387
H	-3.453785	-1.750373	2.445374
H	-2.552062	-2.823215	1.370932
H	-1.737764	-2.075071	2.748137
H	-3.181850	0.691550	2.358443
H	-1.522176	0.407893	2.883313
H	-1.839432	1.448646	1.492793
C	5.534026	-1.297617	-1.207347
C	4.228249	-1.036503	-0.694383
C	3.132101	-1.834023	-1.016483
C	4.043565	0.082703	0.178118
C	2.773850	0.348781	0.686690
C	1.679851	-0.448705	0.365234
C	1.866387	-1.564207	-0.508026
C	6.584367	-0.502362	-0.877576
C	5.173502	0.893451	0.499014
H	2.635740	1.198048	1.347189
C	0.373370	-0.180202	0.878632
C	0.726771	-2.364645	-0.820292
C	-0.494177	-2.070294	-0.310570
C	-0.704186	-0.945933	0.556302
H	0.269218	0.679826	1.527704
H	0.857562	-3.219252	-1.473679
H	-1.333001	-2.705828	-0.564983
C	6.400709	0.612012	-0.009921
H	5.668687	-2.146241	-1.867435
H	3.269447	-2.682597	-1.678074
H	7.570088	-0.710102	-1.273105
H	5.029352	1.740583	1.158939
H	7.249732	1.234642	0.240612

14. References

1 Stanton, M. P.; Hoover, J. M. Copper-Catalyzed Decarboxylative Elimination of Carboxylic Acids to Styrenes. *J. Org. Chem*. **2023**, *88*, 1713-1719.

2 Shimomaki, K.; Murata, K.; Martin, R.; Iwasawa, N. Visible-Light-Driven Carboxylation of Aryl Halides by the Combined Use of Palladium and Photoredox Catalysts. *J. Am. Chem. Soc*. **2017**, *139*, 9467-9470.

3 Lux, M.; Klussman, M. Additions of Aldehyde-Derived Radicals and Nucleophilic *N*-Alkylindoles to Styrenes by Photoredox Catalysis. *Org. Lett*. **2020**, *22*, 3697-3701.

4 Zhang, X.; Liang, N.; Ruining, L.; Sun, Z. Application of Halogen-Bonding Catalysis for Markovnikov-Type Hydrothiolation of Alkenes. *Synlett* **2023**, *34*, 379-387.

5 Hirano, T.; Kamiike, R.; Yuki, T.; Matsumoto, D.; Ute, K. Determination of monomer reactivity ratios from a single sample using multivariate analysis of the 1H NMR spectra of poly[(methyl methacrylate)-co-(benzyl methacrylate)]. *Polym. J.* **2022,** *54*, 623-631.

6 Pracht, P.; Bohle, F.; Grimme, S. Automated exploration of the low-energy chemical space with fast quantum chemical methods. *Phys. Chem. Chem. Phys.* **2020**, *22*, 7169−7192.

7 Bannwarth, C.; Ehlert, S.; Grimme, S. GFN2-xTB-An accurate and broadly parametrized self-consistent tight-binding quantum chemical method with multipole electrostatics and density-dependent dispersion contributions. *J. Chem. Theory Comput.* **2019**, *15*, 1652−1671.

8 Gaussian 16, Revision C.01, M. J. Frisch, G. W. Trucks, H. B. Schlegel, G. E. Scuseria, M. A. Robb, J. R. Cheeseman, G. Scalmani, V. Barone, G. A. Petersson, H. Nakatsuji, X. Li, M. Caricato, A. V. Marenich, J. Bloino, B. G. Janesko, R. Gomperts, B. Mennucci, H. P. Hratchian, J. V. Ortiz, A. F. Izmaylov, J. L. Sonnenberg, D. Williams-Young, F. Ding, F. Lipparini, F. Egidi, J. Goings, B. Peng, A. Petrone, T. Henderson, D. Ranasinghe, V. G. Zakrzewski, J. Gao, N. Rega, G. Zheng, W. Liang, M. Hada, M. Ehara, K. Toyota, R. Fukuda, J. Hasegawa, M. Ishida, T. Nakajima, Y. Honda, O. Kitao, H. Nakai, T. Vreven, K. Throssell, J. A. Montgomery, Jr., J. E. Peralta, F. Ogliaro, M. J. Bearpark, J. J. Heyd, E. N. Brothers, K. N. Kudin, V. N. Staroverov, T. A. Keith, R. Kobayashi, J. Normand, K. Raghavachari, A. P. Rendell, J. C. Burant, S. S. Iyengar, J. Tomasi, M. Cossi, J. M. Millam, M. Klene, C. Adamo, R. Cammi, J. W. Ochterski, R. L. Martin, K. Morokuma, O. Farkas, J. B. Foresman, and D. J. Fox, Gaussian, Inc., Wallingford CT, 2016.

9 Zhao, Y.; Truhlar, D. G. The M06 suite of density functionals for main group thermochemistry, thermochemical kinetics, noncovalent interactions, excited states, and transition elements: two new functionals and systematic testing of four M06-class functionals and 12 other functionals. *Theor. Chem. Acc.* **2008**, *120*, 215−241.

10 Weigend, F.; Ahlrichs, R. Balanced basis sets of split valence, triple zeta valence and quadruple zeta valence quality for H to Rn: Design and assessment of accuracy. *Phys. Chem. Chem. Phys.* **2005**, *7*, 3297−3305.
